# Supplementary material for: Molecular and functional profiling of primary normal ovarian cells defines insights into cancer development and drug responses
Source: Mol Ther Oncol. 2024 Nov 8;32(4):200903. doi: 10.1016/j.omton.2024.200903 (PMC11616607; doi:10.1016/j.omton.2024.200903)
Supplement: Document S1. Figures S1–S7 and Table S2 [file mmc1.pdf]

OMTON, Volume *32*

Supplemental information

Molecular and functional profiling of primary normal ovarian cells defines insights into cancer development and drug responses

Emilia Piki, Alice Dini, Frida Rantanen, Franziska Bentz, Lassi Paavolainen, Harlan Barker, Juuli Raivola, Akira Hirasawa, Olli Kallioniemi, Astrid Murumägi, and Daniela Ungureanu

A

Cell line	Source	Histological subtype	Status	Sample type	Key oncogenic aberrations	HR-status (*BRCA1/BRCA2*)
HOSE1C	Tashiro Lab (1,2)	Epithelial/mesenchymal (normal tissue)	Non-malignant	Ovarian surface epithelium	CDK4, CyclinD1 overexpression, hTERT	
HOSE2C	Tashiro Lab (1,2)	Epithelial/mesenchymal (normal tissue)	Non-malignant	Ovarian surface epithelium	CDK4, CyclinD1 overexpression, hTERT	
JHOS2	RIKEN	High-grade serous ovarian cancer	Primary	Tissue, ovary	TP53 p.S261_splice, BRCA1 N/A, NF1 p.K798R	*BRCA1* mutation
Kuramochi	JCRB Cell Bank	High-grade serous ovarian cancer	Metastasis	Ascites	TP53 p.D281Y, MYC Amp, KRAS Amp	*BRCA2* mutation
Ovsaho	JCRB Cell Bank	High-grade serous ovarian cancer	Metastasis	Tissue, abdomen	TP53 p.R342X	*BRCA2* homozygous deletion
COV318	ECACC	High-grade serous ovarian cancer	Metastasis	Ascites	TP53 p.I195F	
COV362	ECACC	High-grade serous ovarian cancer	Metastasis	Pleural effusion	TP53 p.Y220C, BRCA1 p.P871fs	*BRCA1* mutation
OV90	ATCC	High-grade serous ovarian cancer	Metastasis	Ascites	TP53 p.S215R, BRAF p.N486-P4990del	
CAOV3	NCI-DTP	High-grade serous ovarian cancer	Primary	Tissue, ovary	TP53 p.Q136*	
OVCAR4	NCI-DTP	High-grade serous ovarian cancer	Metastasis	Ascites	TP53 p.L130V	
OVCAR5	NCI-DTP	High-grade serous ovarian cancer	Metastasis	Ascites	TP53 wt, KRAS p.G12V, CDKN2A homozygous loss	
OVCAR8	NCI-DTP	High-grade serous ovarian cancer	Primary	Tissue, ovary	TP53 p.Y126_K132del, ERBB2 p.G776V	

B

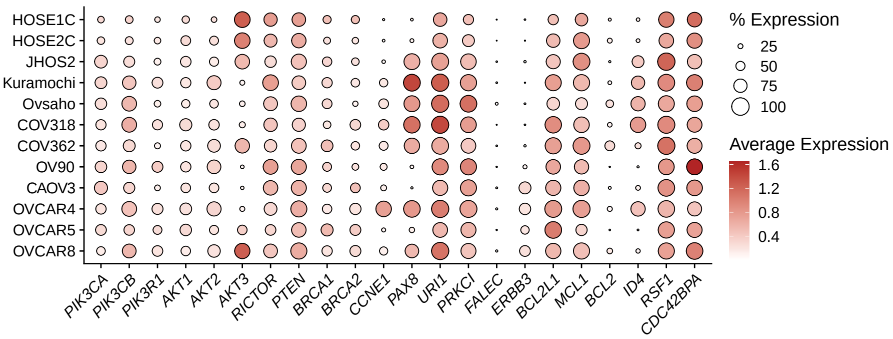

C

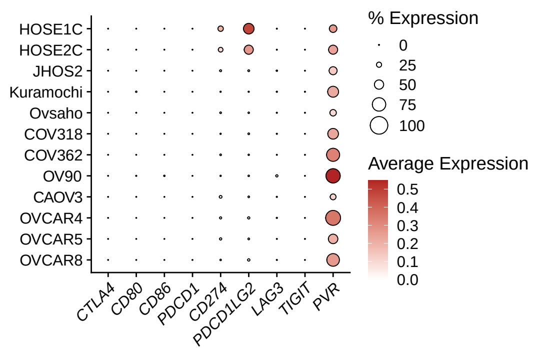

D

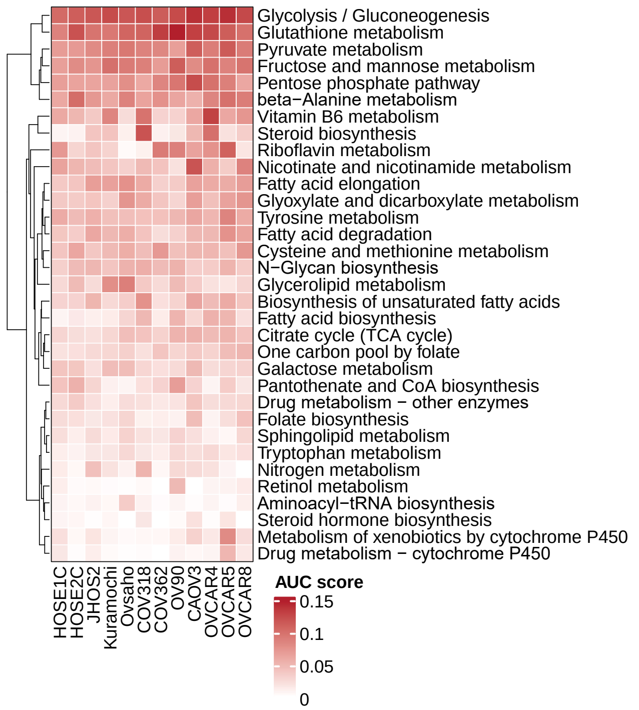

Figure S1

(A) A table presenting the sources of cell lines, their histological subtypes, (disease) status, tissue origin and the key oncogenic aberrations (1 PMID: 15956975, 2 PMID: 19126650).
(B) Expression levels of the relevant targetable genes for two HOSE and ten HGSOC cell lines (PMID: 35582310, PMID: 36804485).
(C) Expression levels of positive immune checkpoints and their ligands for two HOSE and ten HGSOC cell lines (PMID: 22437870, PMID: 23868869).
(D) A heatmap displaying the activity of key metabolic pathways for two HOSE and ten HGSOC cell lines. Pathway activity was quantified using AUC scores.

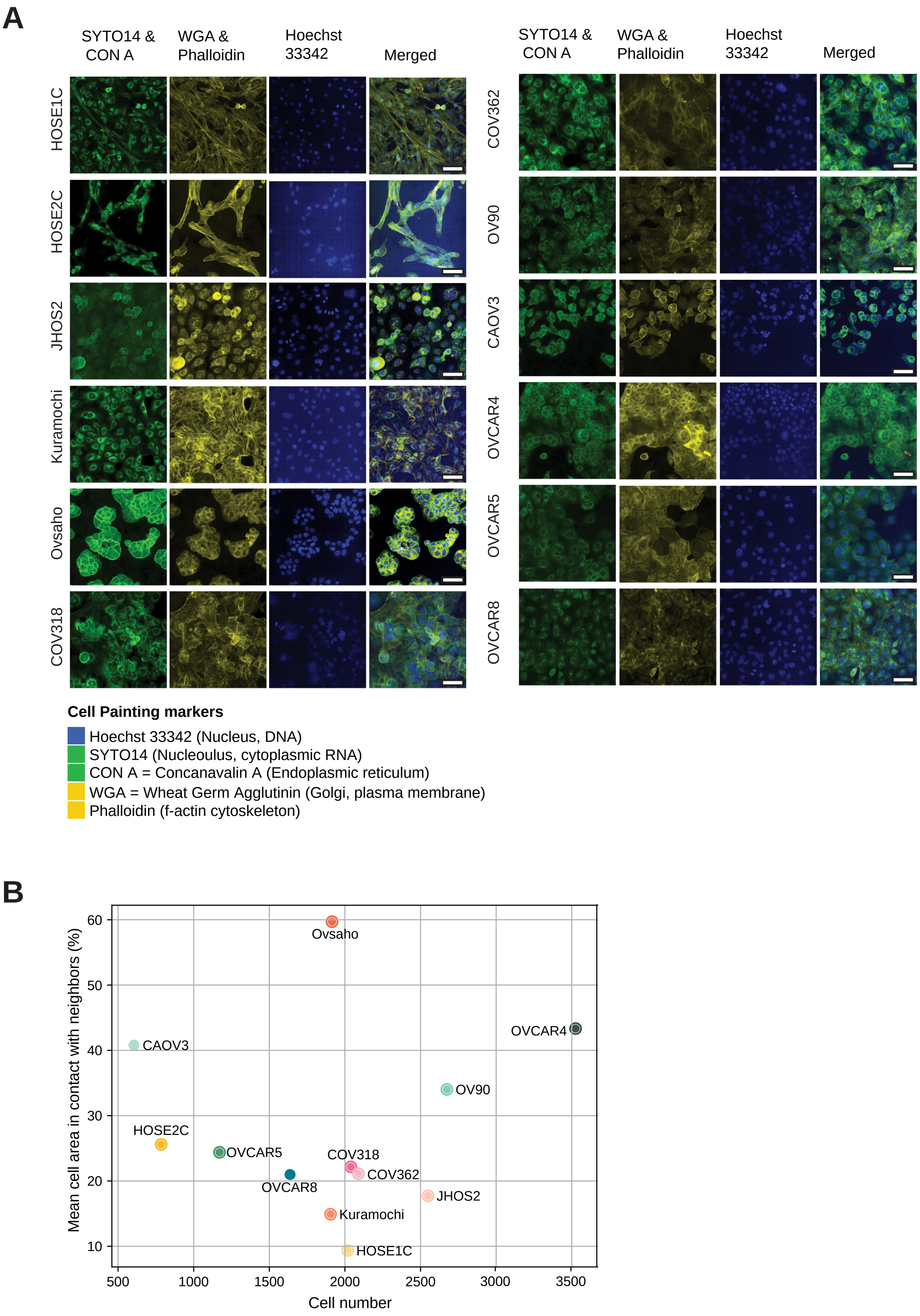

Figure S2

(A) Representative confocal images of Cell Painting of ten HGOC and two HOSE cell lines. The Cell Painting markers used in the assay and the cellular compartments they visualize indicated on the bottom. Scale bar 100 µm.
(B) Relative (%) average mean cell area in contact with neighbor cells based on Cell Painting analysis. X-axis reports average cell number in images.

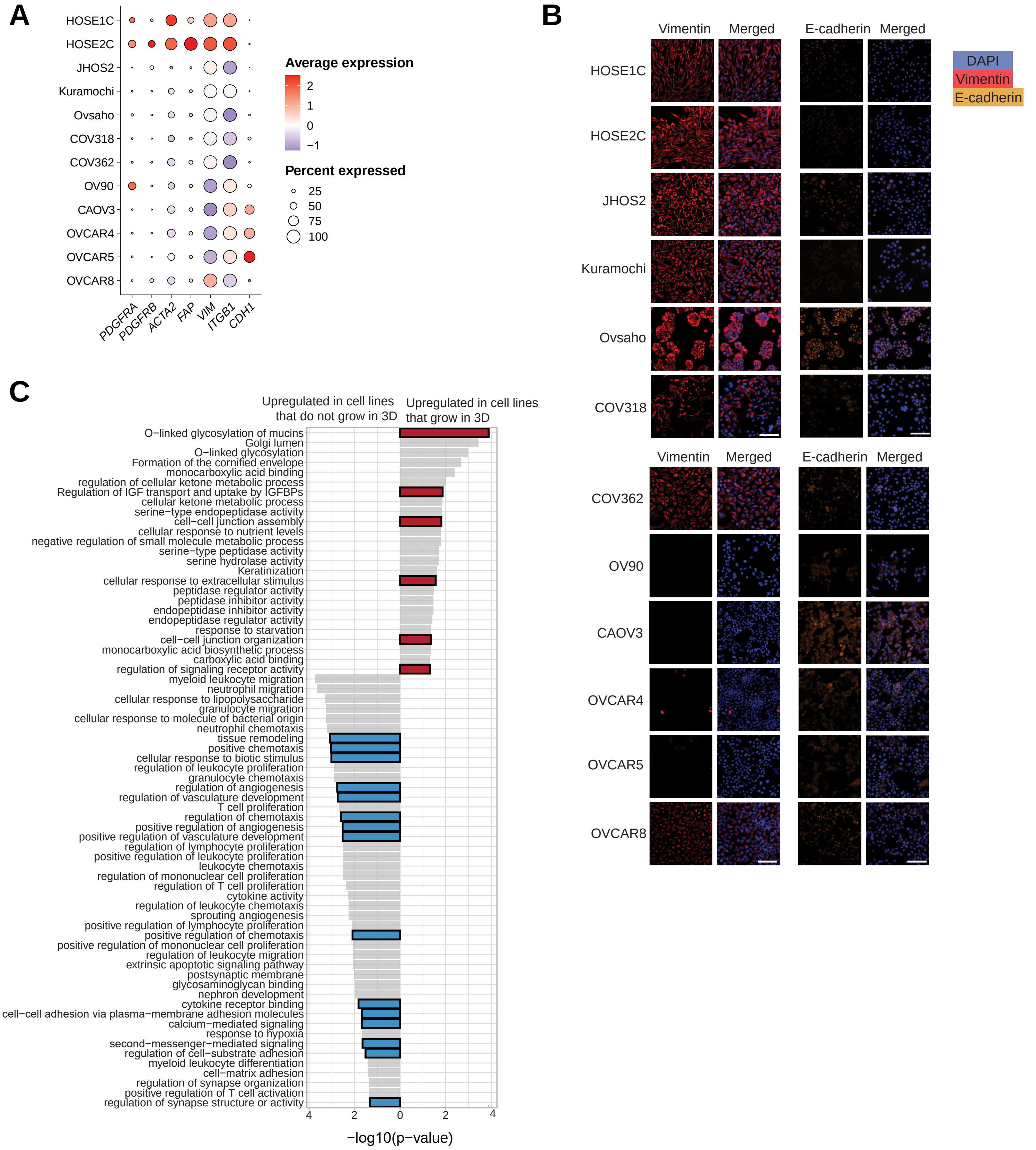

Figure S3

(A) Dot plot illustrating the fraction of cell lines expressing the selected genes (size of dot) and the intensity of expression (color shading). Average expression is scaled.
(B) Immunofluorescence staining of vimentin (in red) and E-cadherin (in orange) of ten HGSOC and two HOSE cell lines. DAPI (in blue) was used as nuclear staining. Scale bar: 100 µm.
(C) Waterfall plot depicting the most upregulated pathways on transcriptomic level in HGSOC cell lines grouped based on their abliity to grow in 3D. Highlighted in colors are pathways selected based on their relevance to OC (related to Figure 3D).

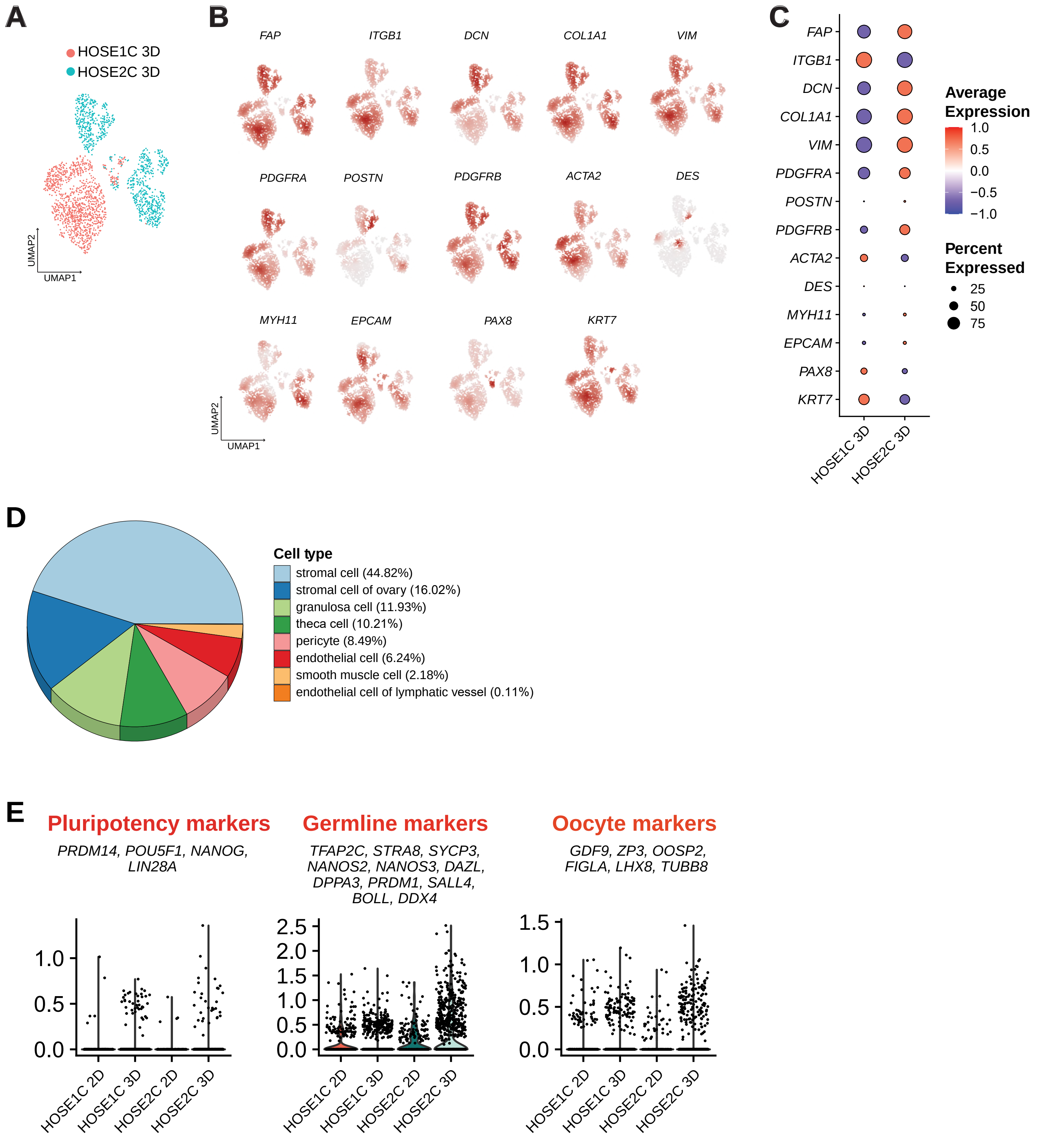

Figure S4

(A) UMAP plot of HOSE1C and HOSE2C cells grown 3D.
(B) UMAP showing the mRNA expression of selected genes in HOSE1C and HOSE2C grown in 3D.
(C) Dot plot illustrating the fraction of cell lines expressing the selected genes (size of dot) and the intensity of expression (color shading).
(D) Pie chart representing the fractions of different ovarian cell subtypes in the Figure 3G, comprising of 39.954 single cells of ovary from CellXGene dataset.
(E) The expression of selected pluripotency, germline and oocyte marker genes in HOSE1C and HOSE2C grown in 2D and 3D.

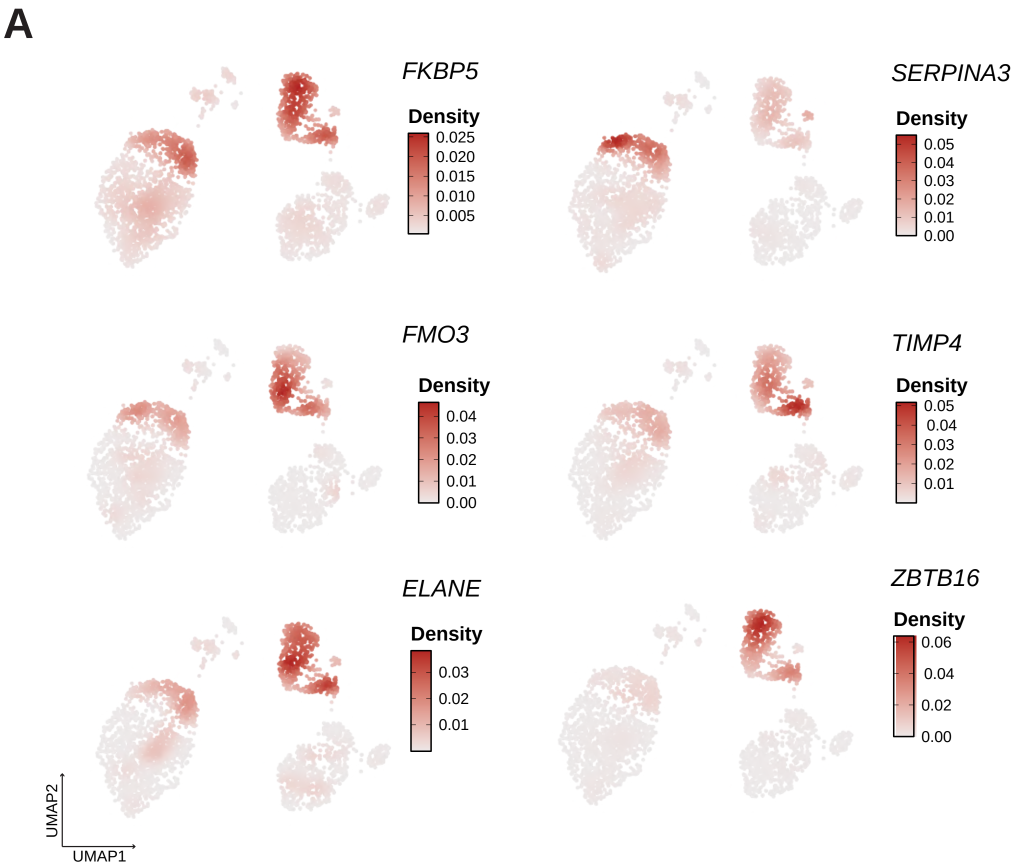

Figure S5

(A) UMAPs showing the mRNA expression of selected genes in untreated and cancer cell- derived condition media (CM) treated HOSE cells in 3D setting. Clustering is presented in Figure 5A.

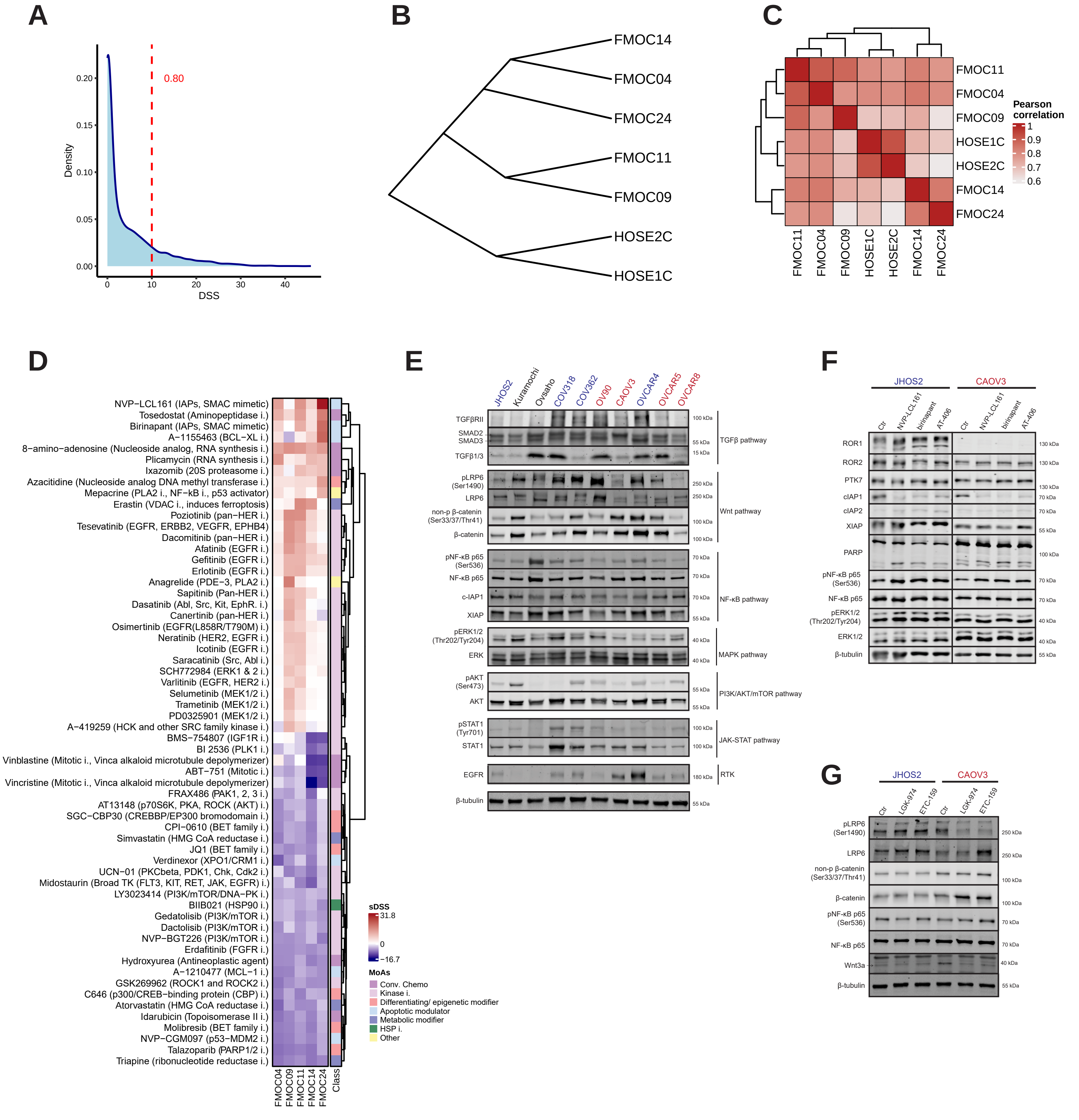

Figure S6

(A) Overall distribution of DSS values across ten OC and two HOSE cell lines. A DSS ≥ 10 (corresponding to the 80th percentile) was chosen as the sensitivity threshold.
(B) Cladogram showing the complete linkage hierarchical clustering results of the five HGSOC PDCs from Murumägi et al., (PMID:36476658) and two HOSEs based on their sensitivity to the 503 drugs included in the DSRT screen.
(C) Pearson correlation among the PDCs and the HOSE cell lines based on their sensitivity to the 503 drugs.
(D) Heatmap showcasing the sDSS for individual drugs. The sDSS was derived by subtracting the average DSS of HOSE1C and HOSE2C from the respective DSSs of PDCs. A subset of the 60 most extreme drugs (top and bottom 30 from the clustered heatmap in Table S1) has been highlighted.
(E) Immunoblot analysis showing the expression of selected markers of TGFβ, Wnt, NF-κB, MAPK, PI3K/AKT/mTOR and JAK-STAT pathways in cell lines lysates.
(F) Immunoblot analysis of JHOS2 and CAOV3 cells treated with SMAC mimetics (NVP-LC161, birinapant and AT-406).
(G) Immunoblot analysis of JHOS2 and CAOV3 cells treated with Porcupine inhibitors (LGK-974 and ETC-159).

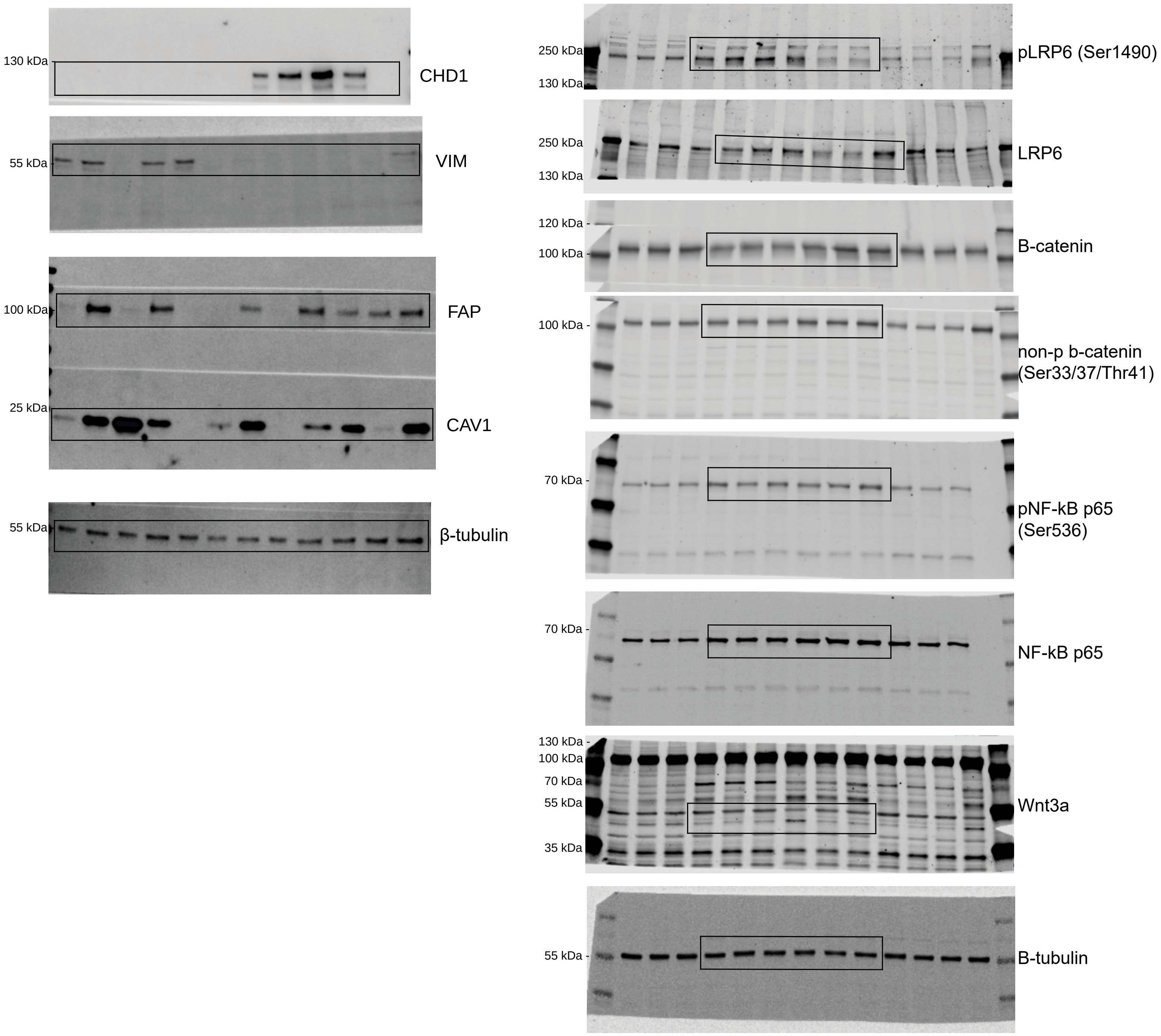

Figure S7

Uncropped images of the immuno blots presented in Figure 3B (left) and in Supplementary Figure 6G (right).

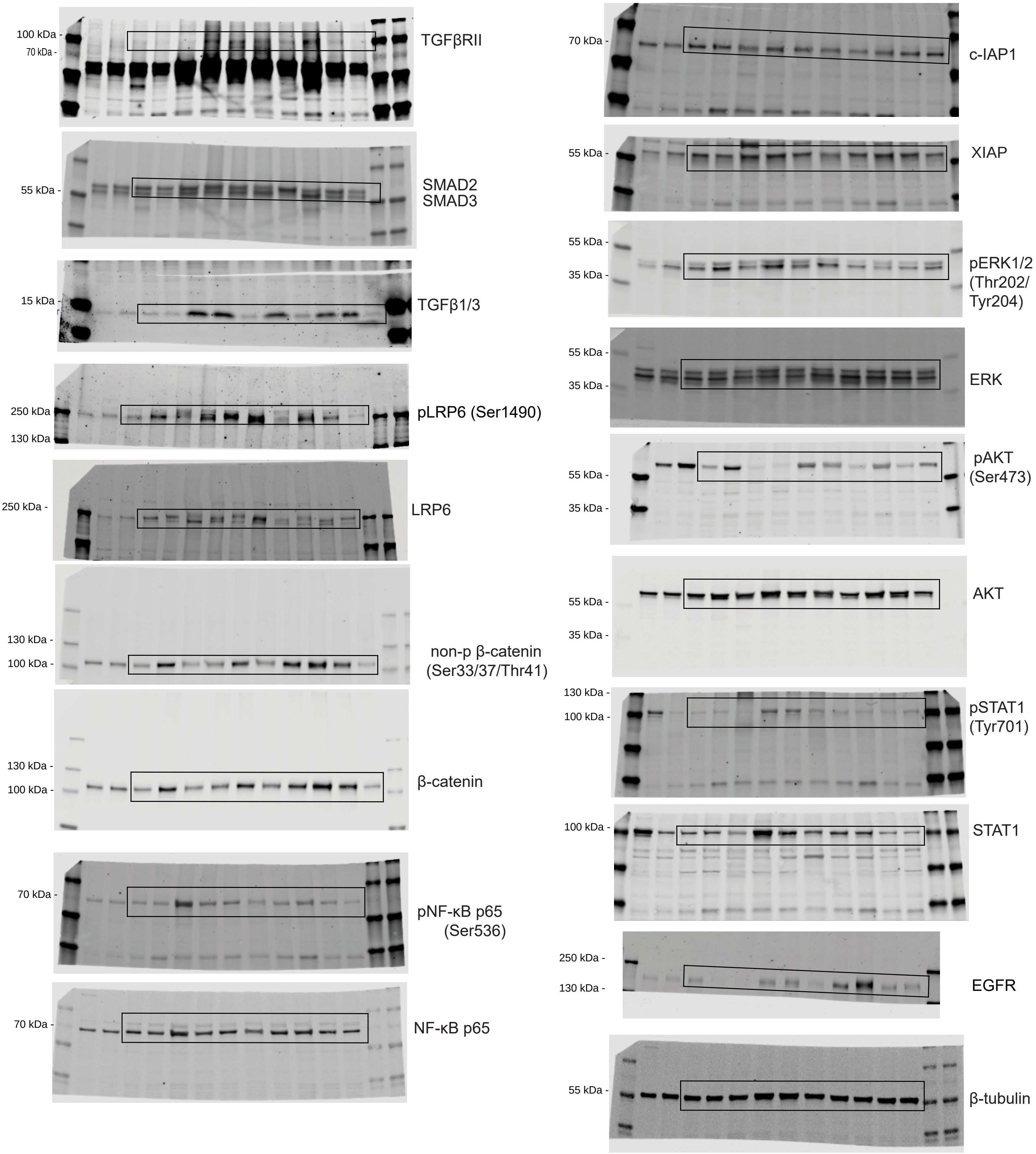

Figure S7

Uncropped images of the immuno blots presented in Supplementary Figure 6E.

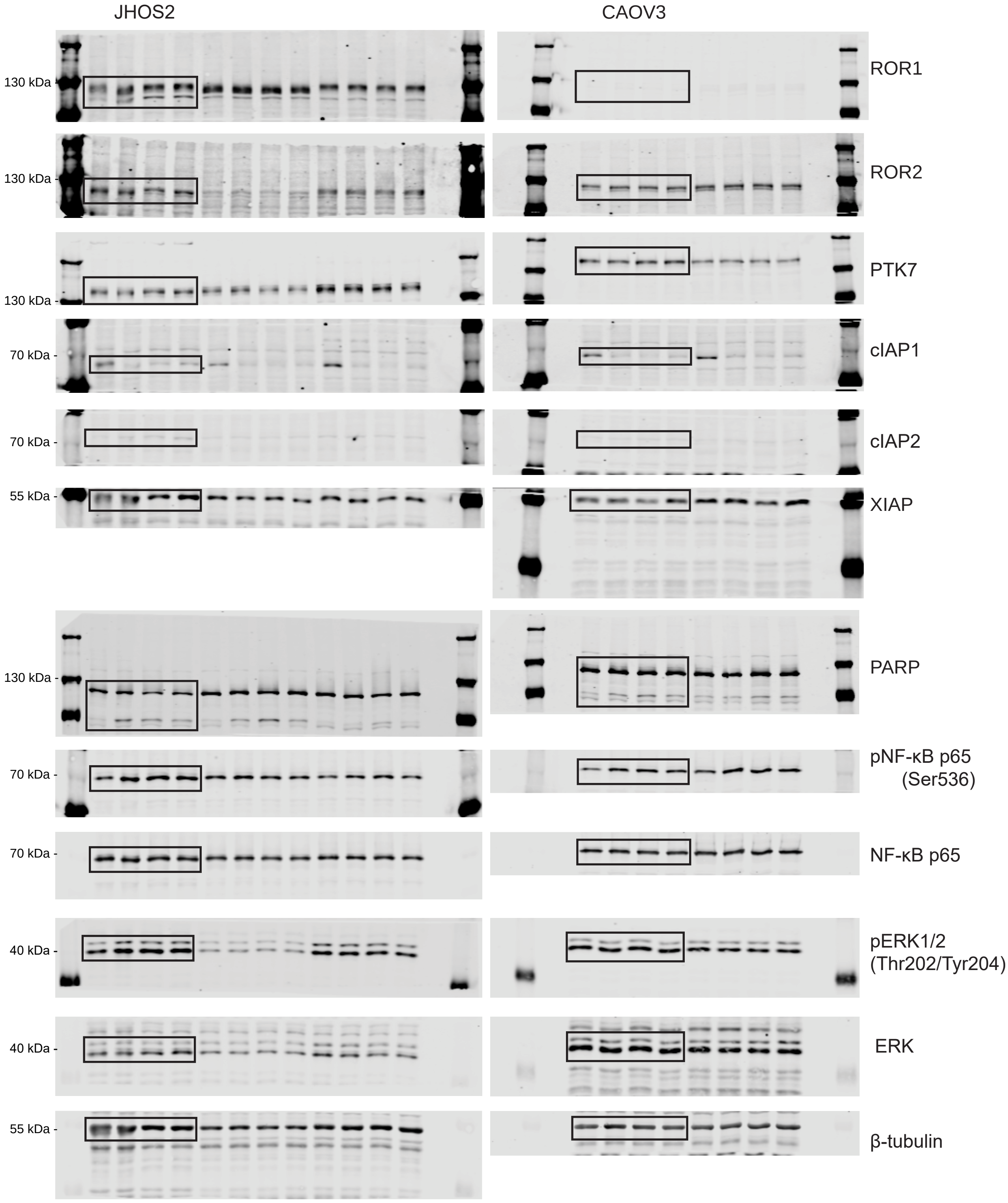

Figure S7

Uncropped images of the immuno blots presented in Supplementary Figure 6F.

Supplemental Tables

Table S1. Drug sensitivity score (DSS) data for all 12 cell lines and 5 PDCs. Data is shown for 10 HGSOC cell lines, 2 HOSE cell lines and 5 patient-derived cancer cells screened with the panel of 503 drugs.

Table S2. Key resources table. For the staining and immunoblotting, the information of the dye and antibody dilutions used in immunofluorescence (IF), immunoblotting (WB) and Cell Painting are indicated in the notes.

REAGENT or RESOURCE	SOURCE	IDENTIFIER	NOTES
Antibodies			
AKT	Cell Signaling Technology (CST)	Cat# 2920; RRID:AB_1147620	WB, 1:1000.
$\beta_2$microglobulin	BioLegend	Cat# 316302; RRID:AB_492835	
β-catenin	Cell Signaling Technology (CST)	Cat# 8480; RRID:AB_11127855	WB, 1:1000.
β-tubulin	Cell Signaling Technology (CST)	Cat# 86298; RRID:AB_2715541	WB, 1:1000.
Caveolin-1 (D46G3)	Cell Signaling Technology (CST)	Cat# 3267; RRID:AB_2275453	WB, 1:1000.
c-IAP1	Cell Signaling Technology (CST)	Cat# 7065; RRID:AB_10890862	WB, 1:1000.
c-IAP2	Cell Signaling Technology (CST)	Cat# 3130; RRID:AB_10693298	WB, 1:1000.
CD298	BioLegend	Cat# 341712; RRID:AB_2876646	
E-Cadherin (24E10)	Cell Signaling Technology (CST)	Cat# 3195; RRID:AB_2893074	IF, 1:100. WB, 1:100.
EGFR	Cell Signaling Technology (CST)	Cat# 4267; RRID:AB_2895042	WB, 1:1000.
ERK	Cell Signaling Technology (CST)	Cat# 4696; RRID:AB_390780	WB, 1:1000.
FAP (EPR20021)	Abcam	Cat# ab207178; RRID:AB_2864720	WB, 1:500.

IRDye® 680RD Donkey anti-Rabbit IgG	LI-COR	Cat# 926-68073; RRID: AB_10954442	
IRDye® 800CW Donkey anti-Mouse IgG	LI-COR	Cat# 926-32212; RRID: AB_621847	
LRP6	Cell Signaling Technology (CST)	Cat# 3395; RRID:AB_1950408	WB, 1:500.
NF-KB p65	Cell Signaling Technology (CST)	Cat# 6956; RRID:AB_10828935	WB, 1:1000.
non-p β-catenin	Cell Signaling Technology (CST)	Cat# 8814; RRID:AB_11127203	WB, 1:1000.
pAKT (Ser473)	Cell Signaling Technology (CST)	Cat# 4060; RRID:AB_2315049	WB, 1:1000.
pERK1/2 (Thr202/Tyr204)	Cell Signaling Technology (CST)	Cat# 4370; RRID:AB_2315112	WB, 1:1000.
PARP	Cell Signaling Technology (CST)	Cat# 9532; RRID:AB_659884	WB, 1:1000.
pLRP6 (Ser1490)	Cell Signaling Technology (CST)	Cat# 2568; RRID:AB_2139327	WB, 1:250.
pNF-KB p65 (Ser536)	Cell Signaling Technology (CST)	Cat# 3033; RRID:AB_331284	WB, 1:1000.
pSTAT1 (Tyr701)	Cell Signaling Technology (CST)	Cat# 88845	WB, 1:1000.
PTK7	Cell Signaling Technology (CST)	Cat# 25618; RRID:AB_2798907	WB, 1:1000.
ROR1	Cell Signaling Technology (CST)	Cat# 16540; RRID:AB_2798764	WB, 1:1000.
ROR2	Cell Signaling Technology (CST)	Cat# 88639; RRID:AB_2800126	WB, 1:1000.
SMAD2/3	Cell Signaling Technology (CST)	Cat# 8685; RRID:AB_10889933	WB, 1:1000.
STAT1	Cell Signaling Technology (CST)	Cat# 65917	WB, 1:1000.
TGFβ1/3	Cell Signaling Technology (CST)	Cat# 3709; RRID:AB_2063357	WB, 1:500.
TGFβRII	Cell Signaling Technology (CST)	Cat# 41896	WB, 1:1000.

Vimentin	Cell Signaling Technology (CST)	Cat# 5741; RRID:AB_10695459	IF, 1:100. WB, 1:1000.
Wnt3a	Cell Signaling Technology (CST)	Cat# 2721; RRID:AB_2215411	WB, 1:500.
XIAP	Cell Signaling Technology (CST)	Cat# 2045; RRID:AB_2214866	WB, 1:1000.
Chemicals, peptides, and recombinant proteins			
Blocker™ BSA	Thermo Fisher Scientific	Cat# 37525	
Concanavalin A	Thermo Fisher Scientific	Cat# C11252	Cell Painting, 1:50, incubation 20min at RT, excitation 488 nm.
DAPI	Sigma-Aldrich	Cat# D9542	
Gibco™ DMEM	Thermo Fisher Scientific	Cat# 41965039	
Gibco™ DMEM/F-12, no glutamine	Thermo Fisher Scientific	Cat# 21331020	
Gibco™ fetal bovine serum	Thermo Fisher Scientific	Cat# 11573397	
Gibco™ HPLM media	Thermo Fisher Scientific	Cat# A4899101	
Gibco™ MEM Non-Essential Amino Acids Solution	Thermo Fisher Scientific	Cat# 11140050	
Gibco™ RPMI 1640	Thermo Fisher Scientific	Cat# 31870025	
Gibco™ TrypLE Express Enzyme	Thermo Fisher Scientific	Cat# 12605010	
Hoechst 33342	Thermo Fisher Scientific	Cat# 62249	Cell Painting, 1:5000, incubation 20min at RT, excitation 405 nm.
L-Glutamine	Sigma-Aldrich	Cat# G7513	
Phalloidin 568	Thermo Fisher Scientific	A12380	Cell Painting, 1:50, incubation 20min at RT, excitation 561 nm.
Primocin®	Invivogen	Cat# ant-pm-2	
Phosphatase Inhibitor Cocktail	Bimake.com	B15001	
SYTO14	Thermo Fisher Scientific	Cat# S7576	Cell Painting, 1:500, incubation 20min at RT, excitation 488 nm.
Triton™ X-100	Sigma-Aldrich	Cat# X100-100ML	

VitroGel ORGANOID-4	TheWell Bioscience	Cat# VHM04-4	
Wheat Germ Agglutinin	Thermo Fisher Scientific	Cat# W32464	Cell Painting, 1:500, incubation 20min at RT, excitation 561nm.
2x Laemmli Sample Buffer	Bio-RAD	Cat# 1610737	
4–20% Mini-PROTEAN® TGX Stain-Free™ Protein Gels	Bio-RAD	Cat# 4568096	
Critical commercial assays			
Chromium Next GEM Single Cell 3' Gene Expression version 3.1 Dual Index chemistry with Feature Barcoding	10x Genomics, FIMM Single-Cell Analytics unit	Cat# CG000317	
CellTiter-Glo® 2.0 Assay	Promega	Cat# G9242	
Deposited data			
DSRT data	This paper		
Experimental models: Cell lines			
COV318	European Collection of Authenticated Cell Cultures (ECACC)	RRID:CVCL_2419	
COV362	European Collection of Authenticated Cell Cultures (ECACC)	RRID:CVCL_2420	
HOSE1C	Tashiro Lab	N/A	
HOSE2C	Tashiro Lab	N/A	
JHOS2	Riken BioResource Research Center (BRC)	RRID:CVCL_4647	
Kuramochi	Japanese Collection of Research Bioresources (JCRB)	RRID:CVCL_1345	
CAOV3	National Cancer Institute - Developmental Therapeutics Program (NCI-DTP)	RRID:CVCL_0201	
OVCAR4	National Cancer Institute - Developmental Therapeutics Program (NCI-DTP)	RRID:CVCL_1627	

OVCAR5	National Cancer Institute - Developmental Therapeutics Program (NCI-DTP)	RRID:CVCL_1628	
OVCAR8	National Cancer Institute - Developmental Therapeutics Program (NCI-DTP)	RRID:CVCL_1629	
Ovsaho	Japanese Collection of Research Bioresources (JCRB)	RRID:CVCL_3114	
OV90	American Type Culture Collection (ATCC)	RRID:CVCL_3768	
Software and algorithms			
QuPath (0.4.3)	Bankhead et al	RRID:SCR_018257	
Image Studio™ Lite (5.2)	LI-COR	RRID:SCR_013715	
ZEN Digital Imaging for Light Microscopy	Carl Zeiss AG	RRID:SCR_013672	
R	R Core Team	RRID:SCR_001905	
CIDRE	Smith et al	N/A	
nucleAIzer	Hollandi et al	N/A	
Cell Profiler (4.2.5)	Stirling et al	RRID:SCR_007358	
Cell Ranger (7.1.0)	10x Genomics	RRID:SCR_017344	
Seurat (5.0.1)	Hao and Hao et al	RRID:SCR_016341	
InferCNV package (1.18.1)	Tickle et al	RRID: SCR_021140	
gprofiler2 package (0.2.2)	Kolberg et al	RRID:SCR_018190	
decoupleR (2.8.0)	Badia-i-Mompel et al	N/A	
PROGENy	Schubert et al	N/A	
pySCENIC (0.12.1)	Aibar et al	RRID: SCR_017247	
BREEZE	Potdar et al	N/A	
GraphPad Prism (9.3.1)	GraphPad Prism Software, LLC	RRID: SCR_002798	
Harmony (0.1.1)	Korsunsky et al	RRID:SCR_022206	
Leiden algorithm	Traag et al	N/A	
