# Supplementary material for: Molecular and functional profiling of primary normal ovarian cells defines insights into cancer development and drug responses
Source: Mol Ther Oncol. 2024 Nov 8;32(4):200903. doi: 10.1016/j.omton.2024.200903 (PMC11616607; doi:10.1016/j.omton.2024.200903)
Supplement: Document S2. Article plus supplemental information [file mmc3.pdf]

Molecular Therapy
Oncology
Original Article

Molecular and functional profiling of primary normal ovarian cells defines insights into cancer development and drug responses

Emilia Piki,[1] Alice Dini,[1] Frida Rantanen,[1] Franziska Bentz,[2] Lassi Paavolainen,[2] Harlan Barker,[1,3] Juuli Raivola,[4] Akira Hirasawa,[5] Olli Kallioniemi,[2,6] Astrid Murumägi,[2] and Daniela Ungureanu[1,2]

[1]Disease Networks Unit, Faculty of Biochemistry and Molecular Medicine, University of Oulu, 90014 Oulu, Finland; [2]Institute for Molecular Medicine Finland (FIMM), Helsinki Institute of Life Science (HiLIFE), University of Helsinki, 00014 Helsinki, Finland; [3]Tampere University Hospital and Faculty of Medicine and Health Technology, Tampere University, 33014 Tampere, Finland; [4]Applied Tumor Genomics, Research Program Unit, Faculty of Medicine, University of Helsinki, 00014 Helsinki, Finland; [5]Department of Clinical Genomic Medicine, Okayama University Graduate School of Medicine, Dentistry and Pharmaceutical Sciences, Okayama, Japan; [6]Science for Life Laboratory (SciLifeLab), Department of Oncology and Pathology, Karolinska Institutet, 171 65 Solna, Sweden

Patients with ovarian cancer, especially the high-grade serous ovarian cancer (HGSOC) subtype, face poor prognosis due to late diagnosis and treatment resistance. Owing to the high heterogeneity of HGSOC, identifying the origin of the disease and optimal treatments is difficult. Here, we characterized two primary immortalized human ovarian cell lines, human ovarian surface epithelium (HOSE)1C and HOSE2C, comparing their molecular profiling with representative HGSOC cells. We identified molecular features associated with normal and malignant phenotype of ovarian cells by applying single-cell transcriptomics, high-content image-based cell painting, and high-throughput drug testing. Our findings reveal distinct transcriptomic and morphological profiles for the two HOSEs, with a stromal phenotype. Moreover, their responses to the tumor microenvironment differ, exemplified by *STAT1* and *GREM1* upregulation in HOSE1C and HOSE2C, respectively. We identified selective activation of ERK/MEK targeted inhibitors in cancer cells compared to HOSEs. This study offers insights into the normal and malignant ovarian cells, shedding light on cancer development and drug responses.

INTRODUCTION

Ovarian cancer (OC) is the most lethal gynecological malignancy and accounts for 5% of cancer deaths in women.[1] High-grade serous OC (HGSOC) is the most common histological subtype diagnosed in more than 70% of patients with OC.[2] Over 75% of HGSOC patients present an advanced disease with widespread metastases and ascites.[3] The molecular stratification of HGSOC shows an extensive heterogeneity characterized by ubiquitous *TP53* mutations, cell-cycle deregulation via *RB1* and *FOXM1* aberrations, homologous recombination deficiency (HRD) present in approximately half of all HGSOCs, and recurrent high-level copy-number amplifications (CNAs).[4–6] While treatment regimens combining platinum-based chemotherapy with bevacizumab have exhibited an extension in 5-year survival rates, the overall clinical advantages are limited.[7] Therefore, improving the therapeutic outcome of HGSOC remains a high priority, focusing on enhancing our understanding of disease pathology, prioritizing translational research tools and models, and developing more effective strategies for the detection and treatment of this challenging cancer.

As a heterogeneous disease, the evolution of cell states and cell types in OC is very complex. The majority of HGSOCs originate from the fallopian tube epithelial (FTE) cells, frequently leading to the identification of malignant cells that disseminate into the fluids accumulating in the peritoneal cavity (ascites) or the pleural effusions of the lungs, indicative of late-stage extra-abdominal metastases.[8] However, other studies have shown that the ovarian surface epithelium (OSE), a layer of epithelial and mesothelial cells covering the ovary, is considered to be the tissue of origin of many subtypes of OC,[9] including HGSOC. As there is no consensus regarding the tissue-of-origin of OC, key questions that remain to be addressed include the biological processes underlying pathogenic development and their impact on treatment outcome in OC.

Here, we applied molecular profiling to normal and malignant ovarian cells combined with functional studies. Our analyses encompassed single-cell RNA sequencing (scRNA-seq) from 2D and 3D models under conditions mimicking normal and tumor-like microenvironment (TME), multiplexed high-content image-based Cell Painting, long-term 3D growth assays, and high-throughput drug sensitivity and resistance testing (DSRT) utilizing a library of 503 drugs with high clinical relevance. We identified distinct transcriptomic, morphological, and drug-response phenotypes associated with the primary hTERT immortalized human ovarian HOSE1C

Received 1 May 2024; accepted 1 November 2024;
https://doi.org/10.1016/j.omton.2024.200903.

Correspondence: Daniela Ungureanu, Disease Networks Unit, Faculty of Biochemistry and Molecular Medicine, University of Oulu, 90014 Oulu, Finland.
E-mail: daniela.ungureanu@oulu.fi

Published by Elsevier Inc. on behalf of The American Society of Gene and Cell Therapy.
This is an open access article under the CC BY license (http://creativecommons.org/licenses/by/4.0/).

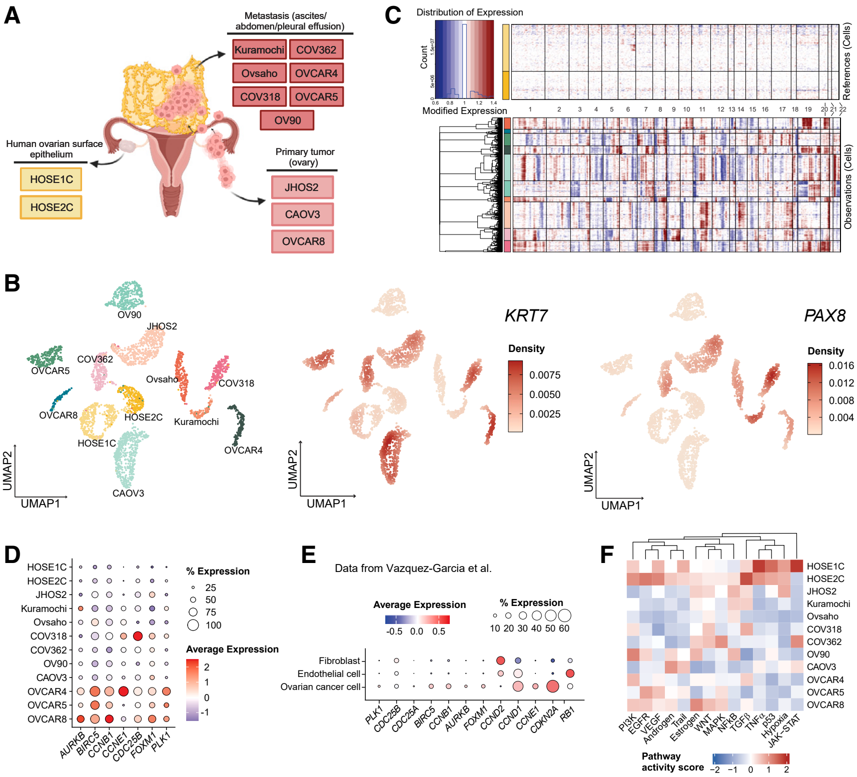

Figure 1. Transcriptome analyses at single-cell level of representative non-malignant and malignant ovarian cell lines

(A) Schematic representation of the origin sites of HGSOC and HOSE cell lines selected for the study. (B) Left: uniform manifold approximation and projection (UMAP) plot of the 10 HGSOC and two HOSE cell lines. Center and right: mRNA expression of *KRT7* and *PAX8* presented as gene-weighted kernel density from log-normalized counts. (C) Inferred CNAs defined for the 10 HGSOC cell lines relative to two HOSE cells. Color annotations represent cell lines as in (B). (D and E) Dot plot illustrating the fraction of cell lines or patient-derived cells expressing the selected genes (size of dot) and the intensity of expression (color shading). The expression is reported in (D) as the scaled average of log-normalized counts. (F) Heatmap of pathway activity scores for PROGENy pathways in 10 HGSOC and two HOSE cell lines.

and HOSE2C cells and show that these cells have a stromal phenotype, whereas it was previously assumed that human ovarian surface epithelia (HOSEs) are epithelial cells.[10] The comparison with single-cell datasets of ovarian tissue provides a glimpse of the cellular heterogeneity and the cell-type-specific dysregulation involved in malignant transformation. We further identify the activation of *STAT1* and *GREM1* in HOSE1C and HOSE2C, respectively, following the exposure of these normal ovarian cells to the TME. Our study revealed inherent distinctions in the phenotypic properties across normal and malignant ovarian cell subtypes while providing a rich resource to explore the heterogeneity and identity of ovarian cells in greater depth.

RESULTS

scRNA-seq transcriptome profiling of non-malignant and malignant ovarian cell lines

To investigate the cellular heterogeneity of normal and malignant ovarian cells, we selected two primary hTERT immortalized human non-malignant OSE cells, HOSE1C and HOSE2C,[10] and 10 representative HGSOC cell lines (Figure 1A). Both HOSEs originate from the normal epithelial and mesenchymal ovarian cell surface layer[10] and harbor a diploid genome without chromosomal instability. The malignant cell lines have been chosen to illustrate HGSOC representability,[11,12] including cells established from primary tumors (JHOS2, CAOV3, and OVCAR8) and metastatic tumors (Kuramochi, Ovsaho, COV318, COV362, OVCAR4, and OVCAR5) covering genomic heterogeneity such as *TP53* and *BRCA1/2* mutations, along with other genomic aberrations (Figure S1A). Our scRNA-seq analysis revealed 12 distinct clusters corresponding to each cell line, with HOSEs clustering proximately, aligning with our expectations (Figure 1B). Variable expression of the cancer proliferative marker cytokeratin 7 (*KRT7*)[13] and the ovarian Müllerian marker paired-box 8 (*PAX8*)[14] were observed across HGSOC cell lines. Moreover, in contrast to the genomic stability observed in HOSEs cells, cancer cells displayed significant heterogeneity following inferCNV[15] analysis of CNAs from scRNA-seq data (Figure 1C). Furthermore, transcriptomic analysis of *FOXM1* signaling networks, a frequently deregulated pathway in 84% of HGSOC tumors,[4] unveiled the downregulation of *CCNB1*, *FOXM1*, and *CCNE1* in HOSEs compared to HGSOC cells (Figure 1D), and the same trend was observed in the normal versus malignant patient samples from Vázquez-García et al. (Figure 1E).[5] Also, we observed variable expression of the most relevant targetable amplified genes in HGSOC cells compared to HOSEs (Figure S1B), especially for phosphatidylinositol 3-kinase (PI3K)-related genes. Next, we employed PROGENy[16] to elucidate the scale of pathway activation in all cells. The analysis showed higher activation of intracellular signaling pathways, including JAK-STAT and tumor necrosis factor alpha (TNFα) in HOSE1C, whereas transforming growth factor beta (TGFβ) was more active in HOSE2C compared to all HGSOC cell lines (Figure 1F). Interestingly, a fraction of HOSEs expressed PD-L2 (Figure S1C), whereas a variable expression of CD155, a member of the nectin family modulating immune function by interacting with receptors expressed by immune cells,[17] was found in all models. In terms of metabolic profiling, we observed lower expression of glycolysis/gluconeogenesis and tricarboxylic acid (TCA)-cycle pathways in HOSEs compared to several of the HGSOC cell lines, although a significant variability in the expression of other metabolic pathways was noticed (Figure S1D).

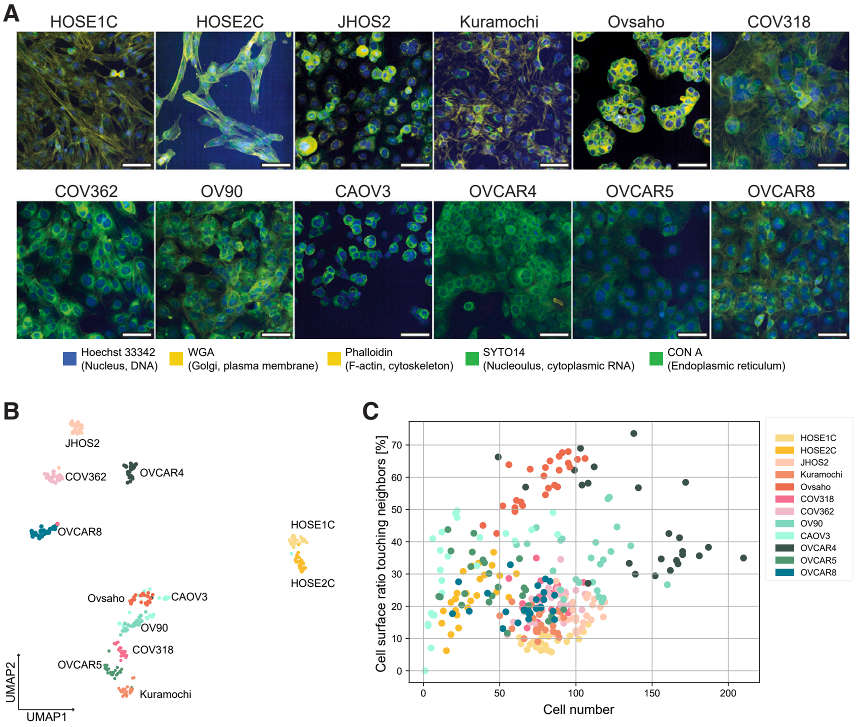

Figure 2. Cell Painting analysis of morphology and growth patterns of ovarian cell lines

(A) A representative Cell Painting image for each of the cell lines. Cell Painting markers used in the assay and the cellular compartments they visualize are indicated under the image panels. Scale bar, 100 μm. (B) UMAP of the top 223 Cell Painting imaging features with the highest variance. Each point represents the average cell number per well. (C) Relative area of total cell surface in direct contact with the cell surface of neighboring cells. Each dot represents the mean value of all cells in one field of view (field of view, 25/cell line).

Morphological profiling with cell painting of HOSEs and HGSOC cells

Next, we analyzed the morphology of HOSEs and HGSOC cell lines using high-content imaging and Cell Painting, an assay that captures features of cells based on microscopy using five stains of cell and organelle components: Hoechst 33342 (DNA), wheat germ agglutinin (WGA; Golgi and plasma membrane), concanavalin A (CONA; endoplasmic reticulum), SYTO 14 (nucleoli and cytoplasmic RNA), and phalloidin (actin) (Figures 2A and S2A). We generated Cell Painting data from all 12 cell lines leveraging a systematic workflow to ensure cells were treated in identical fashion across all rounds of imaging. Morphological profiles were extracted using CellProfiler[18] for image processing, yielding 607 morphological features per cell, followed by downstream analysis of these features. Cell Painting images of the labeled cellular structures showed a distinct phenotype of HOSEs, revealing an elongated cell shape with long actin fibers, typically observed in fibroblast-like cells, especially for HOSE2C (Figure 2A). Uniform manifold approximation and projection (UMAP) analysis based on mean morphological features of cells in fields of view shows that both HOSEs clustered together and separately from all other 10 HGSOC cell lines (Figure 2B). Scatterplot representation of cell neighborhood features based on field of view showed that cell lines such as Ovsaho, CAOV3, OVCAR4, and OV90 have higher percentages of cell surface in contact with neighbors (>30%), whereas the rest of the cell lines have lower percentages (Figures 2C and S2B). This would indicate that these cells maintain a strong adhesion with neighbor cells and have high cadherin-integrin signaling and EMT phenotype, as previously suggested.[19] On the other hand, JHOS2, Kuramochi, OVCAR8, and HOSE cells that have lower percentages of cell surface ratio touching the neighbor cells are likely of epithelial phenotype.

EMT phenotype defines the 3D growth of normal and malignant ovarian cells

Next, we evaluated gene expression in scRNA-seq data of all 12 cell lines of EMT markers *VIM* and *CDH1*, as well as *FAP*, *CAV1*, *ITGB1*, *ACTA2*, *PDGFRA*, and *PDGFRB* representing a group of genes previously associated with the mesenchymal subtype of HGSOC and defining a stromal-like transcriptomic signature.[20] Our results showed strong expression of *FAP* and *VIM*, particularly in HOSE2C (Figure 3A), and both HOSEs exhibited higher *ACTA2*, *PDGFRA*, *PDGFRB*, and *ITGB1* expression compared to HGSOC cell lines (Figure S3A). On the other hand, a subset of HGSOC cells including JHOS2, Kuramochi, and Ovsaho showed high expression of *VIM*, *FAP*, and *CAV1*, while others such as OV90, CAOV3, OVCAR4, and OVCAR5 were *CDH1* positive, indicative of an EMT-like phenotype (Figures 3A and S3A). We verified the transcriptomic findings by western blotting (WB) and immunofluorescence (IF) analyses showing strong VIM, CAV1, and FAP expression in HOSEs cell lysates (albeit stronger in HOSE2C) and high CDH1 levels in OV90, CAOV3, OVCAR4, and OVCAR5 cell lysates (Figures 3B and S3B). These results corroborated cell-painting analyses where CAOV3, OVCAR4, and OV90 exhibited higher cell contact with neighbors than JHOS2, Kuramochi, and HOSEs.

To gain further insights into the transformative potential of both normal and malignant ovarian cell lines, we investigated the ability of cells to sustain long-term growth within a solid matrix, monitoring the development of 3D spheroids over a span of 14 days, followed by quantitative analysis. The findings revealed that five cell lines (HOSE1C, HOSE2C, JHOS2, Kuramochi, and Ovsaho) did not exhibit significant 3D growth, while seven other cell lines (COV318, COV362, OV90, CAOV3, OVCAR4, OVCAR5, and OVCAR8) demonstrated the formation of substantial 3D spheroids (Figure 3C). These observations align with the *VIM/CDH1* expression profiles, indicating a propensity for spheroid formation in *CDH1*-positive cells. Indeed, previous studies have demonstrated that

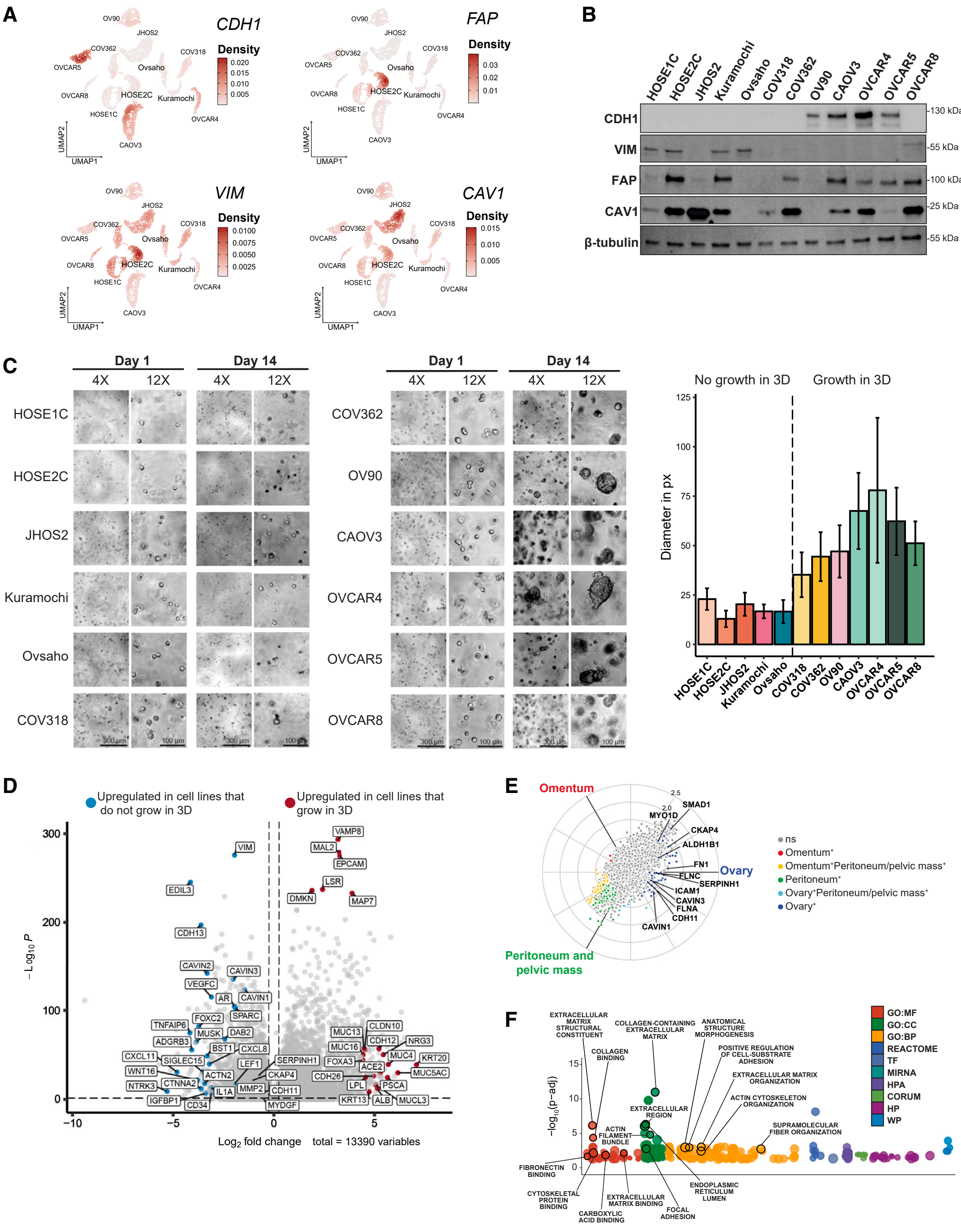

Figure 3. The expression of EMT markers vimentin and E-cadherin determines the phenotypic output regulating 3D growth

(A) UMAPs showing the mRNA expression of selected gene markers in the 10 HGSOC and 2 HOSE cell lines. Color shading indicates the gene-weighted kernel density from log-normalized counts. (B) Immunoblot analysis showing the expression of EMT markers E-cadherin (CDH1), vimentin (VIM), FAP, and caveolin-1 (CAV1) in cell lines lysates. β-Tubulin was used as loading control. (C) Left: representative brightfield images of cell lines spheroids embedded in the hydrogel (Vitrogel), at the starting (day 1) and ending (day 14) points. Scale bars, 300 μm for 4× images and 100 μm for 10× images. Right: average diameter lengths of the day-14 spheroids, measured in pixels. Grouping for the subsequent analyses is indicated in the graph. (D) Volcano plot reporting the distribution of gene-expression fold changes and adjusted *p* values for the comparison of cell

(legend continued on next page)

EMT-high HGSOC tumors have high invasion ability and are linked to poor survival.[21]

We then zoomed into the single-cell transcriptome profile of cell lines to unravel the underlying mechanisms governing 3D growth. A differential gene-expression (DGE) analysis of the scRNA-seq data revealed significant upregulation of *VIM*, *CDH13*, caveolae-related *CAVINs*, *WNT16*, *ACT2*, and *CTNNA2* in cell lines that do not grow in 3D (Figure 3D). Moreover, upregulation of mucin-family genes (*MUC4*, *MUC16*, *MUC13*, *MUCL3*) along with *FOXA3* and *EPCAM* was observed in cell lines that grow in 3D. Furthermore, functional enrichment analysis identified key pathways associated with EMT processes, including regulation of cell substrate adhesion and cell-cell junction organization characterizing the cell lines exhibiting stronger 3D growth. Conversely, pathways such as tissue remodeling, regulation of chemotaxis, and modulation of signaling receptor activity were enriched in cells lacking 3D growth (Figure S3C).

Next, we sought to understand the biology driving HGSOC at the tissue-specific level by looking at the protein expression of various tumor biopsies from Hu et al.[22] An analysis of differential protein expression in samples originating from ovaries, omentum, and peritoneum or pelvic mass identified statistically significant overexpression of CAVIN1, CAVIN3, cadherin 11 (CDH11), filamin A (FLNA), filamin C (FLNC), and SERPINH1 in ovaries, underpinning a functional enrichment related to extracellular matrix, collagen binding, and cytoskeleton signaling (Figures 3E and 3F). This biologically significant information could explain why genes related to caveolae and those driving cytoskeleton signaling, such as vimentin, actins, and cadherins, are involved in phenotypic outputs in cells that are less likely to undergo EMT and 3D growth.

The two primary non-malignant ovarian HOSE cell lines are phenotypically different

Since the two HOSE cell lines showed transcriptomic and morphological differences, we analyzed them separately. Using scRNA-seq data, we investigated the expression of genes involved in stromal-like signature (*FAP*, *VIM*, and *ITGB1*); cancer-associated fibroblasts (CAFs; *POSTN* and *ACTA2*); as well as *COL1A1*, *SULF1*, *TCF21*, and *DLK1*, which were previously associated with FAP-high or FAP-low CAF phenotypes in OC.[23,24] Our results showed higher expression of *FAP*, *ITGB1*, *DCN*, *COL1A1*, and *VIM* in HOSE2C in comparison with HOSE1C, which expressed more *ACTA2* and *KRT7* (Figure 4A). These findings were corroborated by the DGE and functional enrichment analyses of HOSEs scRNA-seq data showing a significant upregulation of several collagen-related genes, *FN1*, and *FBN2* in HOSE2C, whereas genes involved in the interferon (IFN) pathway were significantly enriched in HOSE1C (Figures 4B and 4C).

Next, we investigated whether HOSEs cultured in 2D and 3D settings exhibit transcriptomic changes that would alter their phenotype. The scRNA-seq analyses indicated that each HOSE clustered separately in 3D and, with the exception of *ITGB1*, the expression of fibroblast markers such as *FAP*, *DCN*, *COL1A1*, and *VIM* was higher in HOSE2C compared to HOSE1C as observed in 2D settings (Figures S4A–S4C). This distinct transcriptomic profile was retained when scRNA-seq data from 2D and 3D were combined, showing that each HOSE clustered separately irrespective of the culture conditions (Figure 4D). However, we observed increased expression of *FAP*, *PDGFRA*, and *PDGFRB* in contrast with decreased *ACTA2*, *ITGB1*, and *VIM* in 3D compared to 2D settings of both HOSEs (Figure 4E). Our results corroborate previous findings showing that OC CAFs grown in 3D have high FAP expression.[24] Next, we deployed SCENIC[25] to unravel cell states by scoring the activity of each regulon in each cell for both HOSEs cultured in 2D and 3D settings. SCENIC revealed a clear shift in the regulome activity of each HOSE defined by the 2D vs. 3D culture conditions, marked by the downregulation of transcription factor (TF) networks related to *CREBs*, *MAZ*, *TFDP1*, *IRF3*, *JUND*, *ETVs*, *BATF3*, and *STAT2* in 3D of both cell lines (Figure 4F). Moreover, the upregulation of two groups of cell-proliferation-related TFs (*ATF*, *FOS*, *JUNB*, and *EGR1*, *NF-KB1*, *ETV*, *BATF3*) were observed in HOSE1C 3D, whereas *MYC*, *ZEB1*, *ELK3*, and *TWIST2* were enhanced in HOSE2C 3D compared to 2D.

We further characterized HOSEs and annotated them according to featured markers and genes that have been previously used to describe ovarian cell populations.[26] Based on CellxGene metadata analysis of ovarian tissue, we identified four main cell subtypes, representing stromal cells, granulosa, theca cells, and pericytes, among others (Figure S4D). Both HOSEs clustered closely to stromal and granulosa cells and showed a strong stromal (*PDGFRA*, *DCN*, *APOE*, and *FHL2*) and perivascular (*MYH11*, *MCAM*, *RGS5*, *RERG6*, and *TAGLN*) signature in 3D and 2D conditions, respectively, with some modest scores in granulocyte (*AMH*, *FOXL2*, *BEX1*, and *FST*) and endocytic (*CD34*, *VWF*, *FLI1*, and *CDH5*) markers (Figures 4G and 4H). On the other hand, no significant expression of pluripotency, germline, and oocyte markers were found in either HOSEs in 2D or 3D settings (Figure S4E). In conclusion, our transcriptomic analyses show that both HOSEs have a stromal-like transcriptional profile that is stronger in HOSE2C compared to HOSE1C.

HOSE1C and HOSE2C exhibit different pathway activation when exposed to a malignant microenvironment

We were interested in observing the changes in transcriptomic profiles of HOSEs following long-term exposure (4 days) to cancer-cell-derived conditioned medium (CM) in 3D conditions. Transcriptomic analyses revealed that CM-treated HOSE1C and HOSE2C

lines grouped based on ability to grow in 3D as presented in (C). Only selected differentially expressed genes with statistically significant fold changes (FDR < 0.05, log2 fold change >0.25 or <−0.25) are colored and labeled. (E) Radial volcano plot illustrating the distribution of protein fold changes plotted on polar coordinates. Labeled points indicate proteins with significant fold change (FDR < 0.05), with colors referring to the sample type(s) where a certain gene was found upregulated. (F) Manhattan plot reporting the significance of the overrepresentation analysis for proteins exclusively upregulated in ovary samples.

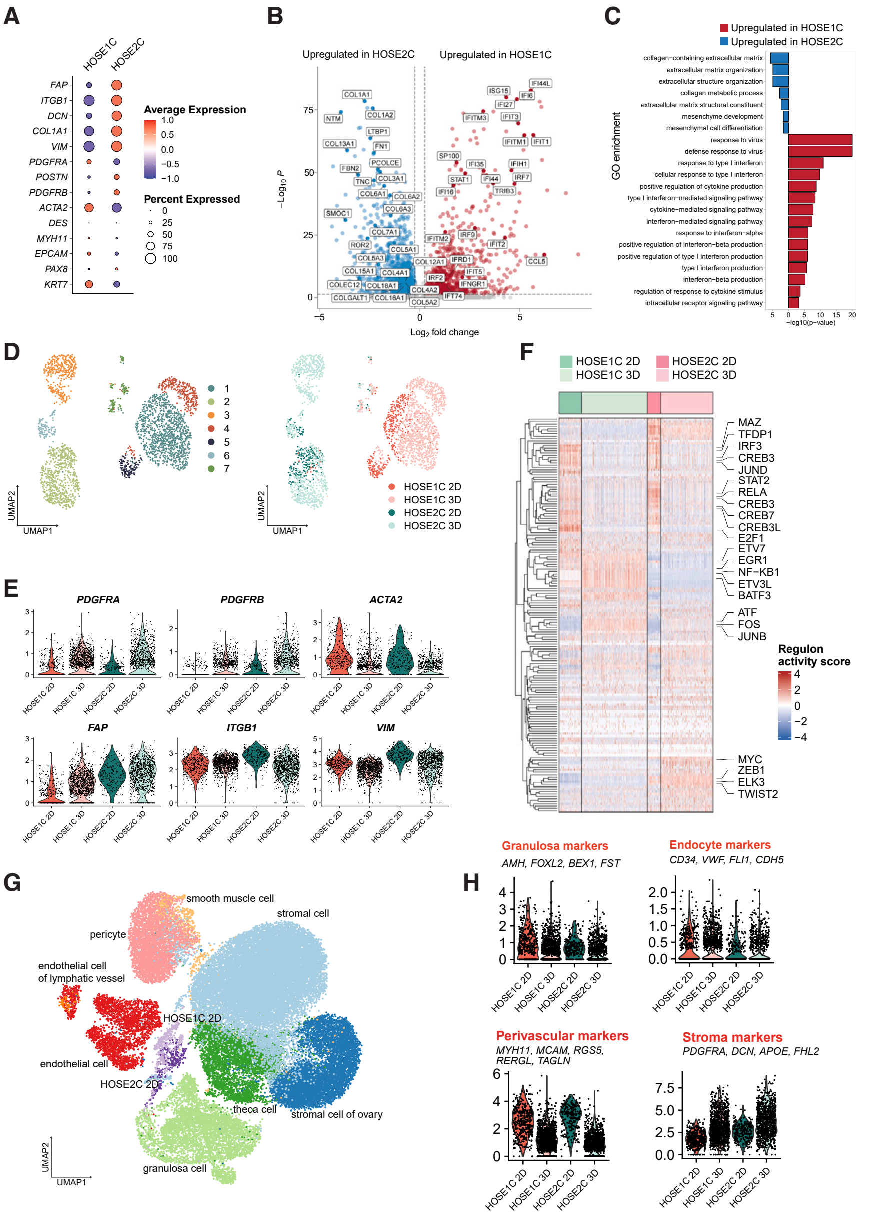

Figure 4. Transcriptomic profiling reveals differences between HOSE1C and HOSE2C cell lines

(A) Dot plot showing the expression of the genes related to stromal and CAF phenotypes for HOSE1C and HOSE2C grown in 2D settings. The percentage of cells expressing the gene is indicated by the diameter of dot. (B) Volcano plot reporting the distribution of gene-expression fold changes and adjusted p values for the comparison of HOSE1C and HOSE2C grown in 2D settings. Only statistically significant, differentially expressed (FDR $<$ 0.05, log2 fold change $>$0.25 or $<-$0.25) selected genes are labeled. (C) Waterfall plot depicting the most relevant upregulated pathways of the top 100 most up- and downregulated differentially expressed genes in the two HOSE cell lines grown in 2D settings using GO enrichment analysis. (D) UMAP showing the Leiden clustering of HOSE1C and HOSE2C cells grown in 2D and 3D settings. (E) Normalized expression of selected genes in HOSE1C and HOSE2C grown in 2D and 3D. (F) SCENIC analysis indicating shift in regulon activity of HOSE1C and HOSE2C cells grown in 3D compared to 2D. The most relevant altered transcription factor regulomes are shown on the right. (G) CellxGene database analysis of ovarian tissue representing HOSE1C and HOSE2C in relation to other cell populations comprising a total of 39,954 cells. (H) Aggregated normalized expression of selected cell-type marker genes in HOSE1C and HOSE2C grown in 2D and 3D.

clustered together with untreated cells; however, these cells were predominantly found in clusters 1 and 2, respectively (Figure 5A). A DGE analysis between clusters 1 and 4 (HOSE1C) and clusters 2 and 3 (HOSE2C) identified the upregulation of several common genes, highlighted by *FKBP5*, a regulator of stress responses[27]; *FMO3*, an oxygenase with a role in insulin regulation[28]; *ELANE*, a neutrophil elastase with high expression in stromal cells[29]; *SERPINA3*, an inhibitor of serine proteases with high expression in gonadotropin-treated endometrium;[30] *TIMP4*, a tissue inhibitor of metalloproteinases with a role in extracellular matrix (ECM) remodeling[31]; and *ZBTB16*, a TF involved in regulation of lineage-specific target genes (Figure S5A).[32] HOSE cells with high expression of these genes were unresponsive to CM treatment. Moreover, a DGE analysis of untreated vs. CM-treated cells resulted in different significantly upregulated genes in each HOSEs. The upregulation of IFN-related *STAT1*, *ISG15*, and *OAS1* was observed in CM-treated HOSE1C, whereas *ITGA2*, *GREM1*, and *CDH13* were upregulated in CM-treated HOSE2C (Figures 5B and 5C), indicating that each HOSE responds to CM treatment differently depending on its own transcriptomic phenotype. Apart from HOSEs, *STAT1* expression was also enriched in stromal cells of the ovaries, whereas *GREM1* expression was enriched in granulosa cells (Figure 5D). These results were corroborated by PROGENy analyses yielding a higher pathway activity score for mitogen-activated protein kinase (MAPK), PI3K, and vascular endothelial growth factor (VEGF) pathways in CM-treated HOSE2C, underlying an intracellular signaling activation of RTKs (Figure 5E). On the other hand, JAK-STAT pathway activation was evident in CM-treated HOSE1C, in accordance with upregulation of IFN-related signaling.

HOSEs and HGSOC cells exhibit distinct drug-response phenotypes

The intra- and inter-tumor heterogeneity of HGSOC underscores the need for methods that can identify not only the specific drug

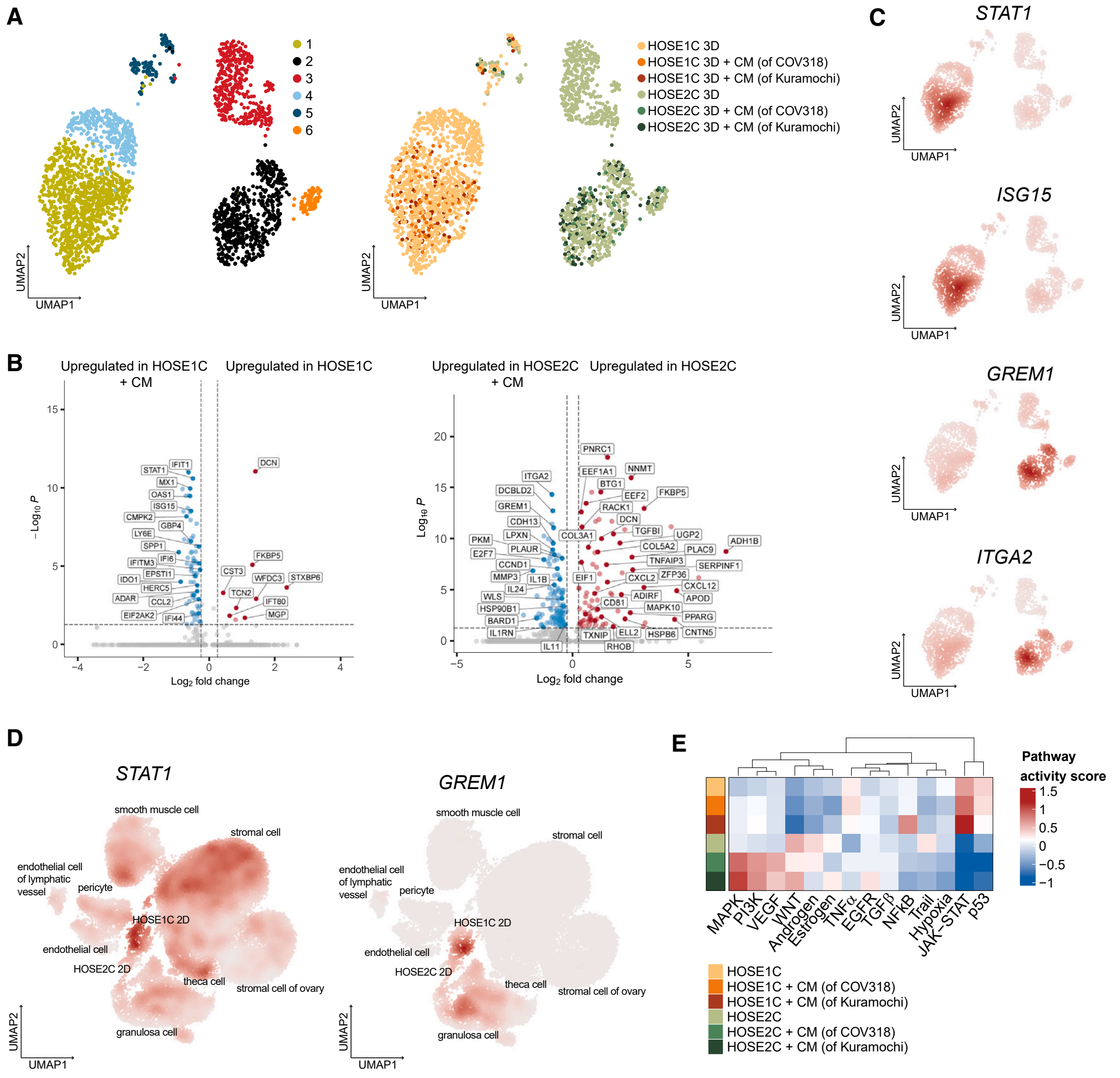

Figure 5. Altered pathway activity of normal ovarian HOSE cells exposed to malignant microenvironment

(A) UMAP showing Leiden and cell-type clustering of untreated and cancer-cell-derived (COV318 or Kuramochi) conditioned medium (CM)-treated HOSE cells in 3D setting. (B) Volcano plot representing the most upregulated differentially expressed genes in HOSE1C and HOSE2C in untreated versus CM-treated cells. (C) UMAPs showing the mRNA expression of selected gene markers in HOSE1C and HOSE2C cells clustered as in (A). Color shading indicates the gene-weighted kernel density from log-normalized counts. (D) UMAP showing *STAT1* and *GREM1* expression in CellxGene database analysis of ovarian tissue including also HOSE1C and HOSE2C cells. (E) Heatmap indicating PROGENy pathway activity scores for untreated and CM-treated HOSE cells in 3D setting.

vulnerabilities of cancer cells but also drug responses in normal cells. To implement this goal, we first subjected all the cell lines to a high-throughput DSRT pipeline. We quantified drug sensitivities of 503 approved and investigational oncology compounds covering chemo- and targeted drugs over a 10,000-fold concentration range (Figure 6A; Table S1) to derive the drug sensitivity score (DSS).[33] We then chose a DSS $\geq$ 10 as a threshold to define the overall drug sensitivity, representing the 80th percentiles of all DSSs (Figure S6A). The non-malignant HOSEs displayed high sensitivity to most drug classes, including kinase inhibitors and chemotherapeutics (Figure 6B). Notably, hierarchical clustering of DSSs identified two distinct groups within HGSOC cell lines: drug resistant (COV318, COV362, OVCAR4, and JHOS2) and drug sensitive (OV90, CAOV3, OVCAR5, and OVCAR8) (Figures 6C and 6D). While HOSEs clustered together and distinctly separate from HGSOC cell lines, their overall DSSs showed a drug-sensitive profile. A differential analysis of DSS to uncover drugs with higher activity in cancer cells compared to non-malignant HOSEs revealed increased DSSs of several kinase inhibitors, including IAP/SMAC mimetics (NVP-LCL161 and birinapant) and ERK/MEKi (trametinib, selumetinib, cobimetinib, ulisertinib, and binimertinib), among others (Figure 6E). Conversely, many chemotherapeutics demonstrated higher DSSs in HOSEs compared to

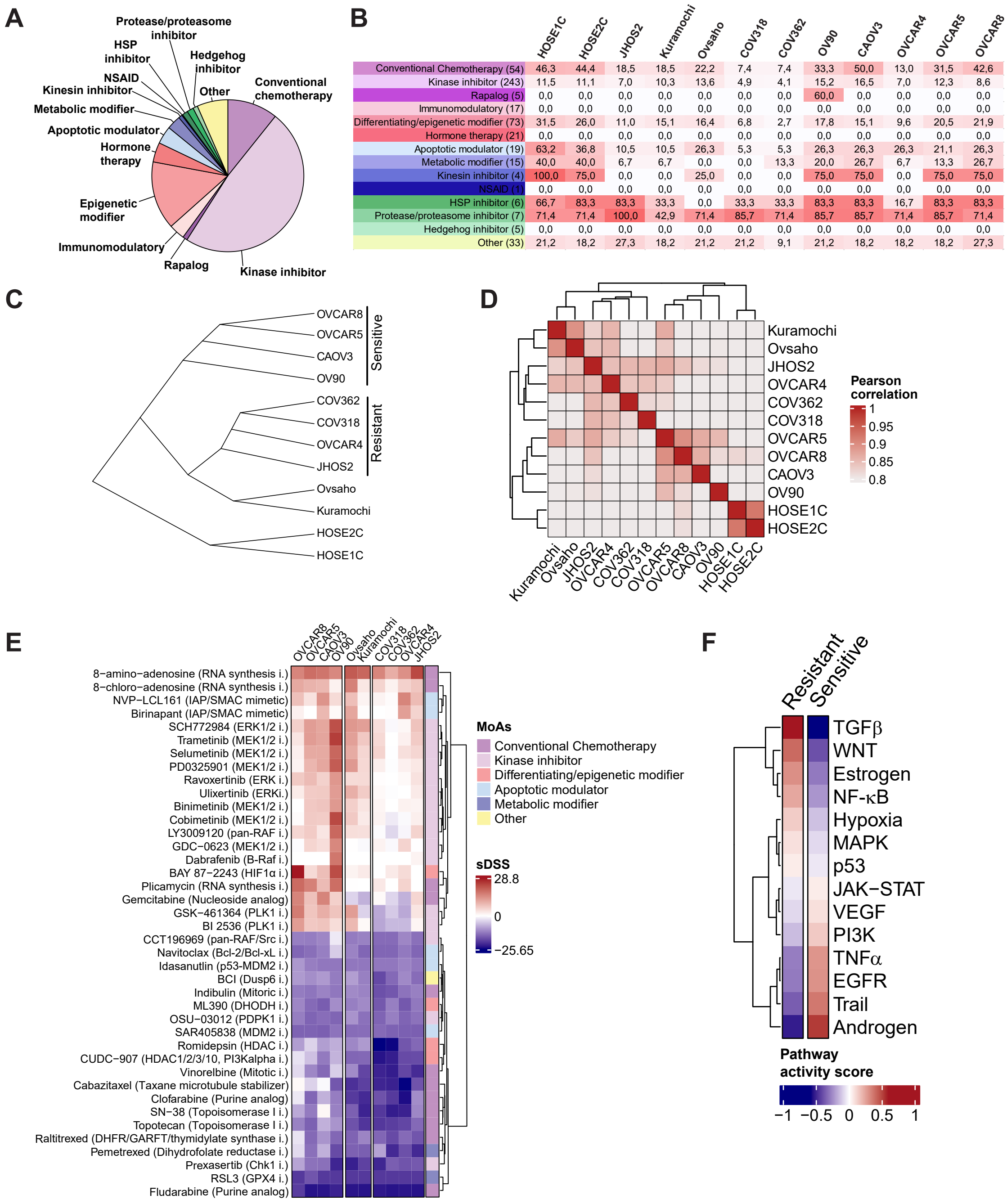

Figure 6. Analyses of drug responses in HGSOC cell lines and non-malignant HOSE cells

(A) Pie chart representing the distribution of the different classes of compounds present in the drug library adopted ($n = 503$). (B) Percentage (%) of effective drugs (DSS $\geq$ 10) presented by drug classes and cell lines. The numbers of drugs in each drug class are indicated in parentheses. (C) Cladogram showing the complete linkage hierarchical clustering results of the 10 HGSOC and two HOSE cell lines based on their sensitivity to the 503 drugs included in the DSRT screen. Grouping (sensitive/resistant) for the subsequent analyses is indicated on the right. (D) Pearson correlation among the cell lines based on their sensitivity to the 503 drugs. (E) Heatmap showcasing the selective drug sensitivity score (sDSS) for individual drugs. The sDSS was derived by subtracting the average DSS of HOSE1C and HOSE2C from the respective DSSs of cell lines. A subset of the 40 most extreme drugs (top and bottom 20 from the clustered heatmap in Table S1) has been highlighted. The color blocks reflect the mechanism of action of the drug used to treat the cells as in (A) and (B). (F) Heatmap illustrating the PROGENy signaling pathway activity scores in sensitive and resistant HGSOC cell lines pooled together.

cancer cells, emphasizing the broad cytotoxic impact of these compounds. Therapeutically relevant, several of HGSOC cell lines and HOSEs were sensitive to cisplatin, a chemo-drug routinely used for OC treatment.

To further validate our findings, we utilized *ex vivo* HGSOC patient-derived cancer cells (PDCs) that were previously published by our group.[34] We compared PDC DSRT profiles with those of our HOSE cells. As anticipated, the HOSE cells clustered separately from the HGSOC PDCs (Figures S6B and S6C), clearly demonstrating that these models exhibit distinct drug-response profiles. Additionally, differential DSS analyses revealed significant differences between HOSEs and PDCs, with the PDCs showing higher sensitivity to several targeted therapies, such as EGFRi and MEKi, while HOSEs demonstrated greater responsiveness to chemotherapeutics (Figure S6D). Conclusively, our results show that HOSEs have distinct DSRT profile characterized by high sensitivity to chemotherapeutics and low response to targeted drugs such as MEKi, when compared to cancer cell lines or *ex vivo* PDCs. Furthermore, we employed PROGENy[16] to elucidate the scale of pathways activation withstanding drug responses in HGSOC cells. We observed higher activation of intracellular signaling pathways, including JAK-STAT, VEGF, EGFR, Trail, and PI3K, in drug-sensitive cells compared to their drug-resistant counterparts, in which TGFβ, WNT, nuclear factor κB (NF-κB), and hypoxia pathways were more activated (Figures 6F and S6E). To validate these findings, we evaluated the effects of inhibitors targeting the Wnt and NF-κB signaling pathways in two HGSOC cell lines representing resistant and sensitive models (Figures S6F and S6G). We observed that SMAC mimetics (birinapant, NVP-LCL161, and AT-406) upregulated ROR1, pNF-κB, and pERK in the drug-resistant JHOS2 cells but not in the drug-sensitive CAOV3 cells, suggesting a role for these targets in drug resistance as previously shown.[35] Similarly, Wnt inhibitors modulated pLRP6/LRP6 expression and increased β-catenin levels in CAOV3-sensitive cells, but not in JHOS2-resistant cells. Together, our heterogeneous panel of cell lines with varied phenotypes enabled a window into the biology of drug responses and unveiled previously undescribed lineage differences that could have implications in understanding the mechanisms of therapeutic agents.

DISCUSSION

Identifying effective therapeutic treatments is a major challenge to improving outcomes for patients with HGSOC, a disease characterized by molecular, cellular, and anatomical heterogeneity at both intra- and inter-patient level.[36] Evidence suggests that HGSOC develops from serous tubal intra-epithelial carcinoma precursor cells and FTE cells that harbor a TP53-related mutational signature.[21] Interestingly, both FTE and OSE cells have been identified as cells of origin for HGSOC in a mouse model,[37] highlighting our incomplete understanding of the underlying mechanisms responsible for OC initiation and progression. Given the phenotypic diversity inherited from the cell of origin, the identification of appropriate cell models that faithfully represent both non-malignant and malignant phenotypes is crucial for functional studies and drug screening.

In this study, we conducted scRNA-seq on primary hTERT immortalized ovarian epithelial HOSE1C and HOSE2C cells alongside 10 representative HGSOC cell lines,[11] aiming to elucidate distinct phenotypes between malignant and non-malignant ovarian cells. The initial molecular characterization of HOSEs assumed that these cells are of epithelial origin, given the anatomic site of their isolation as the OSE.[10] Our deconvolution analyses based on single-cell transcriptomics unveiled different profiles highlighted by strong expression of stromal signatures, with clear differences among the two cell lines. HOSE1C showed high expression of an IFN-related pathway marked by *STAT1*, *IF-*, and *ISRE*-related genes, whereas HOSE2C was characterized by a robust expression of *FAP*, *DCN*, *VIM*, *IGTB1*, *COL1A1*, and *CAV1*, defining a stromal phenotype. We speculate that HOSEs were exposed to external mechano-chemical stimuli that may directly drive their transformation into a fibroblast phenotype, as previously demonstrated.[38] For instance, mechanical stress can induce collagen accumulation in the mesenchyme and make the epithelial cells undergo EMT, which could be the case for HOSE2C. Alternatively, due to mechanical stress, epithelial cells act as targets and producers of immune factors that in turn can induce a mesenchymal phenotype, as could be the case for HOSE1C. Understanding these variations enhances our knowledge of normal ovarian cell biology and may offer insights into how specific epithelial subtypes contribute to ovarian health or disease.

When integrated with the public single-cell transcriptomic datasets of ovarian cells,[26] both HOSEs clustered together and closer to the granulosa and stromal cell types, corroborating their ovarian origin but not epithelial phenotype. These findings suggest that HOSEs may originate from mesenchymal-like ovarian surface cells and have transitioned toward a more fibroblast-like state.[20] A comparison of 2D and 3D models of HOSE transcriptomic profiles showed increased *FAP*, *PDGFRA*, and *PDGFRB* expression in 3D settings for both cell lines, a trait previously demonstrated for fibroblasts grown in 3D.[24] However, we did not observe a CAF-like phenotype defined by the high or low level of FAP expression as suggested by the same study, which indicates that HOSEs are of non-malignant origin.

Conversely, a distinct pattern emerged among HGSOC cells based on *VIM/CDH1* expression profile, with *CDH1*-positive cells demonstrating the most robust 3D growth, indicative of an EMT-like phenotype. Indeed, previous studies have demonstrated that EMT-high HGSOC tumors have high invasion ability and are linked to poor survival.[4,21] While HOSEs did not show 3D growth and transformative potential like several other HGSOC cell lines, our transcriptomic and cell painting analyses showed that HOSEs are not epithelial cells but represent more stromal-like ovarian cell profile.

The distinct phenotype of HOSEs and the underlying differences among the two cell lines prompted us to investigate how these non-malignant ovarian models respond to a tumor-like microenvironment represented by the CM from cancer cells. Our transcriptomic analyses of CM-treated HOSEs uncovered gene-expression profiles defining responses to cancer-like TME, highlighted by *STAT1*,

ISG15, and *OAS1* in HOSE1C and *ITGA2*, *GREM1*, and *CDH13* in HOSE2C. *STAT1* is a TF overexpressed in OC and can act as oncogene or tumor suppressor depending on the disease stage and tissue heterogeneity,[39] whereas *OAS1* was shown to be upregulated in several cancers to promote tumor anti-viral responses.[40] It is plausible that chemokines and other interleukins from CM are likely the activators of IFN-related pathway in HOSE1C, which prompts the hyperactivation of JAK-STAT signaling. On the other hand, *GREM1* upregulation was observed in CM-treated HOSE2C cells. *GREM1/2* are members of the DAN family of BMP inhibitors that are expressed in the ovaries and have roles in regulating later stages of follicle development,[41,42] whereas other studies have found that *GREM1* is predominantly expressed in chicken oocytes and granulosa cells.[43] Indeed, our transcriptomic analyses of ovarian cells identified higher expression of *GREM1* in granulosa cells compared to other ovarian cell subtypes, and its upregulation could likely promote cell proliferation while inhibiting differentiation and steroidogenesis as previously suggested,[43] a trait that can result in malignant phenotype. Furthermore, *ITGA2* was also shown to be involved in regulation of ovarian granulosa cell morphogenesis[44] and could sustain HOSE2C transformation by promoting activation of intracellular MAPK, PI3K, VEGF, and Wnt signaling. The distinct responses of HOSE1C and HOSE2C cells to the TME suggest different functional roles in tumor progression. HOSE1C upregulates IFN-related pathways, potentially promoting anti-tumor immune responses and limiting tumor growth. In contrast, HOSE2C activates RTK-associated pathways, linked to increased proliferation, survival, and migration, which may favor tumor progression. Taken together, our data demonstrates that the response of ovarian cells to TME is specific and regulated by the cell identity, and further studies should delineate the molecular details of these transformations.

The most relevant therapeutic question is whether drug responses differ in the non-malignant HOSEs compared to HGSOC cell lines, as anticipated. Our DSRT screens revealed more similar drug-response profiles in the two HOSEs compared to HGSOC cells, although Pearson correlation analysis highlighted the overall high similarity among all cell lines, likely due to a shared tissue lineage. However, HGSOC cell lines exhibited divergent drug-response phenotypes and were divided into drug-resistant and drug-sensitive groups. Our PROGENy analyses uncovered the activation of JAK-STAT, VEGF, EGFR, and PI3K pathways in drug-sensitive cells, in contrast to TGFβ, WNT, NF-κB, and hypoxia pathways that emerged in drug-resistant group. Our findings align with previous results showing that TGFβ and the hypoxic environment drive tumor progression and drug resistance in multiple cancers, including OC.[45–47] Furthermore, PI3K and WNT were identified among the top six activated cancer pathways in HGSOC, which is significant for disease evolution[48] and therapeutic targeting. Interestingly, our DSRT screens revealed the efficacy of ERK/MEKi (trametinib, selumetinib, cobimetinib, ulisertinib, binimertinib) in HGSOC cell lines (of both sensitive and resistant group) and PDCs but not in HOSEs, and this selective targeting could offer a therapeutic advantage. High MEK/ERK activation is prevalent in HGSOC samples, correlating with poor survival.[49,50] Cisplatin treatment activates the MEK1/2 pathway, leading to enrichment of chemoresistant cancer stem cells with increased MEK1/2 activity.[51,52] Our results suggest that targeting ERK/MEK or their modulators could be an effective strategy in treating HGSOC, either alone or in combination therapies, and underscores the need for biomarkers to guide their use in personalized treatment approaches.

In conclusion, in our study, we present a multi-omics phenotypic characterization of non-malignant and malignant OC preclinical models to aid in functional studies and drug discovery. We show that, while HOSEs are transcriptomically and cytologically different than most of other HGSOC cell lines, these cells resemble the stromal-like phenotype. The fact that HOSEs cells were sensitive to many drug classes suggests that the specificity and cytotoxicity of anti-cancer drugs should be carefully analyzed using representative non-malignant and malignant cellular models.

MATERIALS AND METHODS

Cell culture

OC cell lines were obtained from sources listed in Figure S1. HOSE cell lines[10,53] were received as a gift from Dr. Hironori Tashiro (Department of Gynecology, Faculty of Medical and Pharmaceutical Science, Kumamoto University, Japan).

Cells lines were cultured in their respective standard media. Ovsaho, Kuramochi, CAOV3, OVCAR4, OVCAR5, and OVCAR8 were grown in RPMI 1640 medium (Gibco, catalog # 31870025) supplemented with 10% fetal bovine serum (FBS) and 2 mM L-glutamine (Sigma-Aldrich, catalog # G7513). JHOS2 was grown in DMEM/F-12 medium (Gibco, catalog # 21331020) supplemented with 10% FBS and 1× MEM non-essential amino acid solution (Gibco, catalog # 11140050). COV318, HOSE1C, and HOSE2C were grown in DMEM/F-12 medium (Gibco) supplemented with 10% FBS and 2 mM L-glutamine. COV362 and OV90 were grown in DMEM (Gibco, catalog # 41965039) supplemented with 10% FBS and 2 mM L-glutamine. 0.01% Primocin (InvivoGen, catalog # ant-pm-2) was added to all the media. TrypLE Express (Gibco, catalog # 12605010) was used to detach cells for passage and experiments. For cell counting, TC20 Automated Cell Counter (Bio-Rad) was used. Cell lines were maintained at 37°C with 5% CO_2 and passaged weekly. Cell lines regularly tested negative for *Mycoplasma*.

3D spheroid culture in hydrogel

Cell lines were cultured in 3D using VitroGel ORGANOID-4 hydrogel (TheWell Bioscience, catalog # VHM04-4). For the growth experiment, 5,000 cells were embedded in 66% VitroGel on a 384-well plate (cell-repellent surface) in a volume of 25 μL according to the manufacturer's recommendations. Five replicates were used. The plate was incubated for 15 min at 37°C to ensure hydrogel solidification prior to the addition of 25 μL of medium. Human Plasma-Like Medium (HPLM) (Gibco, catalog # A4899101) supplemented with 5% FBS and Primocin (InvivoGen) was used for all the cell lines to unify the growing conditions and for a more physiologically relevant

environment. Spheroid development was monitored by capturing images with Leica DMi1 microscope equipped with a Flexacam C1 camera, and the top medium was refreshed every 4 days.

Quantification of spheroid diameters from day-14 images was performed using QuPath software (version 0.4.3).[54] To form a representative view of spheroid sizes, 50 individual spheroids were measured, and averages were calculated for each cell line. Data analysis was performed using GraphPad Prism software.

Small-scale drug exposure

JHOS2 and CAOV3 cells were seeded on six-well plates, 300,000 cells per well. After 24-h incubation at 37°C and 5% CO_2, cells were exposed to drugs (1 μM LGK-974, 1 μM ETC-159, 500 nM NVP-LCL161, 100 nM birinapant, 1 μM AT-406) for 24 h, followed by cell lysis and immunoblotting.

Immunoblotting

For immunoblotting, cells were lysed with Triton X-100 lysis buffer (50 mM Tris-HCl pH 7.5, 10% glycerol, 150 mM NaCl, 1 mM EDTA, 1% Triton X-100, 50 mM NaF) supplemented with protease and phosphatase inhibitor cocktails (Bimake, catalog # B15001). Lysates were mixed with Laemmli sample buffer (Bio-Rad, catalog # 1610737), separated in SDS-PAGE, and transferred to nitrocellulose membranes. The primary antibodies and dilutions used for immunoblotting are listed in Table S2. Secondary antibodies were IRDye 800CW Donkey anti-Mouse immunoglobulin (Ig)G or IRDye 680RD Donkey anti-Rabbit IgG (LI-COR, catalog # 926-68073 and catalog # 926-32212) and blots were scanned with Azure 600 (Azure Biosystems). Image analysis was done using Image Studio Lite software (LI-COR). The whole blots for the immunoblots shown in the main figures are shown in Figure S7.

Immunofluorescence

For immunofluorescence assays, 10,000 cells were seeded into a 96-well plate in 100 μL of medium and allowed to grow for 3 days. In the selected wells, cells were fixed with 4% paraformaldehyde for 30 min, followed by washing with PBS. Standard immunofluorescence staining protocol was used. Briefly, permeabilization was performed with 0.1% Triton X-100 in PBS for 10 min, followed by two PBS washes, blocking with 1% BSA (Thermo Fisher Scientific, catalog # 37525) in PBS for 1 h at room temperature (RT), and introduction of primary antibodies overnight at 4°C. Primary and secondary antibodies, and the dilutions used, are listed in the Table S2. Prior to imaging, wells were washed with PBS, and secondary antibodies were applied diluted to 1% BSA in PBS for 1 h at RT. The wells were washed and 2 μg/mL DAPI (Sigma-Aldrich, #D9542) was incubated for 5 min at RT followed by final PBS wash and capturing images with LSM780 Confocal Microscope (Carl Zeiss). Images were exported from ZEN Blue software (Carl Zeiss AG, version 3.1).

DSRT

DSRT was performed as described earlier.[33,34] Briefly, drugs were dispensed on the bottom of 384-well plates using an Echo 550 acoustic dispenser (Labcyte) in dimethyl sulfoxide (DMSO) or water in five different concentrations covering 10,000-fold range. Subsequently, 1,500 cells per well were dispensed on drug plates using the Multidrop dispenser (Thermo Fisher Scientific) followed by 3-day incubation at 37°C and 5% CO_2. Cell viability was measured using CellTiter-Glo Cell Viability Assay (Promega, catalog # G9242) and luminescence signal was measured with PHERAstar FS (BMG Labtech). DSSs were calculated as previously described using the web-based Breeze software[55,56] for each cell line and drug separately. The heatmaps were produced using R (4.3.2) and the package ComplexHeatmap (2.18.0).[57] For PDCs, the threshold for sensitivity was set to 8, which corresponds to the 80th percentile of the DSS distribution.

Cell Painting and analysis

Cell Painting was performed according to Bray et al.,[18] excluding Mito Tracker Deep Red dye. Initially, cells were cultured and fixed on 96-well plate as described in the "immunofluorescence" section. Permeabilization was performed using 0.3% Triton X-100 for 20 min at RT, followed by two PBS washes and addition of staining mix. Hoechst, SYTO14, wheat germ agglutin, concanavalin A and phalloidin dyes were mixed together in suitable dilutions in PBS with 1% BSA 0.1% Tween 20 and added to the wells in a volume of 50 μL for 20 min at RT in the dark. Subsequently, the wells were washed twice with PBS and imaged with Opera Phenix High Content Screening System (PerkinElmer). Both confocal and non-confocal images were taken as 40× images, 25 sites per well.

Illumination correction was done using the CIDRE method[58] and nuclei segmentation was performed with nucleAIzer deep-learning model in BIAS software.[59] Image analysis steps were conducted in CellProfiler (4.2.5).[60] These steps included cell segmentation and feature extraction including measurement of cell neighborhood as a ratio of touching surface pixels to adjacent cells. Additionally, nucleoli were segmented from the Hoechst channel by first enhancing dark holes by an inverted rolling-ball algorithm (EnhanceOrSuppressFeatures module in CellProfiler). This step was followed by nucleoli segmentation from nuclei regions using an adaptive Otsu threshold method using the minimum and maximum diameter of objects as 15 and 40 pixels, respectively. All the remaining parameters in IdentifyPrimaryObjects module were kept as default. The average number of nucleoli per cell was calculated for each cell line. Intensity, shape, and texture were measured for both cells and nuclei. The downstream analysis was performed using the SciPy[61] and scikit-learn[62] Python libraries. Features were selected using the mean values per cell line to determine the features with the highest variance. Plots depicted in this work represent either the mean value per cell line or per field of view of each image.

scRNA-seq

Sample preparation

The HGSOC cell lines, namely Ovsaho, COV318, COV362, OV90, CAOV3, OVCAR4, OVCAR5, OVCAR8, and part of Kuramochi, along with the HOSE cell lines, were collectively processed in one

experimental batch. HOSE cells grown in 3D and HOSE cells incubated in the presence of CM were also processed in separated batches. All the HGSOC and HOSE cell lines, both grown as 2D monolayer and 3D in ultra-low attachment (ULA) plates, were seeded in HPLM supplemented with 5% FBS and Primocin (InvivoGen) prior to scRNA-seq. Live-cell labeling of the three batches was performed as previously described[63] using antibody-oligo conjugates directed against $\beta_2$microglobulin (BioLegend, catalog #316302), and CD298 (BioLegend, catalog #341712).

CM preparation and experimental setup

Kuramochi and COV318 cells were seeded at 1 million cells per mL in HPLM with 5% FBS, and incubated for 72 h, after which the supernatant was harvested, sterile filtered, and stored. HOSE cell lines were seeded at 10,000 cells per well in 96-well ULA plates in HPLM with 5% FBS and incubated for 4 days in the presence or absence of 50% CM, followed by cell harvesting for scRNA-seq analyses.

Library chemistry, sequencing, and raw FASTQ files preprocessing

Single-cell gene-expression profiles were studied using 10x Genomics Chromium Single Cell 3′RNAseq platform (10x Genomics, catalog # CG000317). The Chromium Single Cell 3′RNAseq run and library preparation were done using the Chromium Next GEM Single-Cell 3′ Gene Expression version 3.1 Dual Index chemistry with Feature Barcoding technology. Libraries were sequenced on an Illumina NovaSeq 6000 system (Illumina) using read lengths: 28 bp (read 1), 10 bp (i7 index), 10 bp (i5 index), and 90 bp (read 2). The target minimum coverage was 25,000 reads per cell. Raw FASTQ file preprocessing was performed using Cell Ranger (version 7.1.0, 10x Genomics) pipelines. Specifically, cellranger mkfastq was used to produce FASTQ (raw sequence data) files and cellranger count to perform alignment and UMI counting. Alignment was performed against the GRCh38 (GENCODE v32/Ensembl 98) assembly of the human genome.

scRNA-seq data analysis

The scRNA-seq data bioinformatic analyses were performed using R (4.3.2), primarily utilizing the R package Seurat (5.0.1).[64] First, the hashtag-oligo (HTO) data for the three experimental batches was normalized the function NormalizeData with the centered log-ratio transformation. Then, cells were demultiplexed using the function HTODemux with default parameters. All the negative and doublet cells were discarded. The remaining pool of cells from the HTO classification was subjected to mRNA data quality controls according to the number of UMIs and percentage of UMIs from mitochondrial genes. Normalization and variance stabilization of the HGSOC and HOSE cell lines' batches of scRNA-seq data were performed via NormalizeData and highly variable features were found using FindVariableFeatures using default parameters. Then, the layer data were joined, and the cell cycle heterogeneity was scored with CellCycleScore, using the cell cycle phase references provided by Tirosh et al.[65] Subsequently, the Seurat object was re-split into the original layers' data, and they were scaled by regressing out the G2M and S scores. Principal-component analysis (PCA) followed on the RNA data using RunPCA with default parameters, and with IntegrateLayers. Harmony was chosen as the integration method, and UMAP was computed with RunUMAP using the reduction outcoming from the Harmony integration procedure. Large-scale chromosomal alterations from scRNA-seq datasets were inferred by the inferCNV package (1.18.1).[66] HOSE cells were used as a reference for the CNA inference. The default options were used except for cutoff = 0.1, denoise = TRUE, HMM_type = "i6," and analysis_mode = "sample." The gene order file was created using the script https://github.com/broadinstitute/infercnv/blob/master/scripts/gtf_to_position_file.py downloaded locally and launched on the 10x Genomics genes.gtf file retained within the reference used for the genome alignment as previously described with cellranger. Kernel density estimation plots were produced using the R package Nebulosa (1.12.0)[67] with log-normalized counts (data slot). Differential expression analysis between cell lines that grow in 3D and cell lines that do not grow in 3D was conducted using the FindMarkers function with the log-normalized counts and default settings. The results were filtered to contain only the significant protein coding genes, and the p value was adjusted based on Bonferroni correction. The gene-expression fold changes and adjusted p values for the comparison of cell lines grouped based on ability to grow in 3D were visualized in a volcano plot using the package EnhancedVolcano (1.20.0).[68] Only selected differentially expressed genes with statistically significant fold changes (FDR < 0.05, log2 fold change >0.25 or <-0.25) were colored and labeled. The differentially expressed genes were further ordered based on average $\log_2$ fold change value, and the 100 most up- and downregulated genes were extracted. Functional enrichment analysis was conducted on the resulting gene lists, using the gost function of the gprofiler2 package (0.2.2)[69] and the Reactome and Gene Ontology (GO) databases. The p value was corrected using the FDR method, and terms with an adjusted p value <0.05, term size <300, and intersection size ≥ 5 were retained. The results depicting the most enriched pathways on transcriptomic level of cell lines that grow in 3D and cell lines that do not grow in 3D were visualized in a waterfall plot using the ggplot2 package (3.4.4),[70] highlighting the most relevant pathways. Pathway activity scores at the single-cell level were computed using decoupleR (2.8.0)[71] and the PROGENy[16] model weights of pathways and their target genes. The human weights for each interaction and the top 500 responsive genes ranked by p value were used for inference with the multivariate linear model (run_mlm) following the package's vignette. The results' heatmap was plotted using ComplexHeatmap.

HOSE1C and HOSE2C grown in 2D were pre-processed, UMAP was computed with RunUMAP (dims = 1:15, min.dist = 0.7), and DGE analysis was performed using the FindMarkers function using default settings. The resulting genes were filtered to only contain protein-coding genes. The result was visualized using the EnhancedVolcano package. Functional enrichment analysis was

conducted on the 100 most up- and downregulated genes using the gprofiler2 package (0.2.2) and the GO database. The *p* value was corrected using the FDR method, and the most relevant pathways were visualized in a waterfall plot using ggplot2.

HOSE1C and HOSE2C grown in 2D and 3D were preprocessed and integrated using Harmony integration. The UMAP was computed with RunUMAP (dims = 1:25, min.dist = 0.7), nearest neighbors were computed using the FindNeighbors function (dims = 1:25) followed by clustering using the Leiden algorithm via the FindClusters function, where a resolution of 0.1 was selected. The normalized counts and kernel density estimate of relevant genes were visualized using the VlnPlot function and the Nebulosa package using the data slot. Average expression of relevant genes was visualized using the DotPlot function.

pySCENIC (0.12.1)[25] was used via command-line interface to perform the regulome analysis in HOSE1C and HOSE2C grown in 2D and 3D. The raw UMI count expression matrix was used as input data. We used only the genes that had more than zero count per cell and is detected in at 1 per 1,000 cells in the global dataset, and we excluded both mitochondrial and ribosomal genes. After exporting the Seurat matrix as loom file with build_loom from the R package SCopeLoomR (https://github.com/aertslab/SCopeLoomR, 0.13.0), the loom matrix was used for the gene regulatory network (GRN) inference via pyscenic grn with default parameters. Next, the genes available in the cisTarget human hg38 motif databases were used (https://resources.aertslab.org/cistarget/databases/homo_sapiens/hg38/refseq_r80/mc9nr/gene_based/) to define the modules of TF regulons with pyscenic ctx adding the parameter –mask_dropouts to the default ones. Finally, the regulon modules score for each cell were calculated with pyscenic aucell, again with default parameters. The results were re-integrated back into Seurat and plotted in a heatmap using ComplexHeatmap. Aggregated normalized counts of selected granulosa, endocyte, perivascular, pluripotency, germline, oocyte, and stroma marker genes[26] in the subset were visualized in violin plots using the VlnPlot function.

HOSE1C and HOSE2C grown in 3D were preprocessed, UMAP was computed with RunUMAP (dims = 1:15, n.neighbors = 10, min.dist = 0.9), FindNeighbors (dims = 1:15) was applied, and the data were clustered using FindClusters with the Leiden algorithm. Kernel density estimation of relevant genes was performed using Nebulosa. HOSE1C and HOSE2C grown in 3D and in CM were preprocessed, RunUMAP was performed with specified parameters (dims = 1:25, min.dist = 0.7), FindNeigbors (dims = 1:25) was applied, the data were clustered using the Leiden algorithm in the FindClusters function, and resolution 0.1 was selected. Kernel density estimates for relevant genes were visualized using Nebulosa with the data slot. Pathway activity scores were computed using decoupleR as previously mentioned. The differentially expressed genes were filtered to contain only protein-coding genes and were visualized using the EnhancedVolcano package.

CellxGene database search

Meta data annotations and mRNA counts for approximately 63 million human cells were retrieved from the CellxGene database[72] using the CellxGene-census Python library (1.10.2). Cells were filtered to "tissue" annotation of "ovary," "assay" annotation of "10x" and "cell_type" corresponding to blood cells were removed, resulting in a final count of 39,954 cells. Proportions of cell_type annotations for this final cohort of cells were plotted as a pie chart using the pie3D function from the plotrix package (3.8–4).[73] The counts data were split into layers based on their batch of origin and merged with the dataset containing HOSE1C and HOSE2C cells grown in 2D medium. Normalization and variance stabilization were conducted using the NormalizeData function, followed by identification of highly variable features through the FindVariableFeatures function with default parameters. Scaling of the data was applied, and dimensionality reduction was performed using the RunPCA function with default parameters. The data were integrated using the IntegrateLayers function with the Harmony method, with parameter "groups" set to batch of origin. The UMAP was computed using the RunUMAP function with specified parameters (dims = 1:15, n.neighbors = 15, min.dist = 0.9), based on the Harmony reduction. The kernel density estimates of relevant genes were visualized using the Nebulosa package.

DATA AND CODE AVAILABILITY

All data relevant to the study are included in the article or uploaded as supplemental information and are available on reasonable request from the corresponding author.

ACKNOWLEDGMENTS

We thank Dr. Antti Hassinen, Mariliina Arjama, Dr. Hanna Karvonen, Salla Hyyppä, and Wilhelmiina Niininen for excellent technical assistance. We thank the Biocenter Finland-supported FIMM High-Throughput Biomedicine Unit for providing pre-plated drug plates, FIMM High Content Imaging and Analysis Unit for providing the high-content confocal imaging services, and FIMM Sequencing Unit for providing the sequencing services. Furthermore, these operations thank the Biocenter Finland for instrument funding. This work was supported by the University of Oulu and funded by Academy of Finland (Profi6 #336449, #333583, #288475, and #271845 to D.U.; #349787 to J.R.; and #340273 to L.P.); Sigrid Juselius Foundation and Finnish Cancer Foundation (to D.U. and O.K.); Oulu University UniOGS/HBS-DP to E.P.; and University of Helsinki DPBM to F.B. The authors wish to acknowledge the CSC-IT Center for Science (Finland) for computational resources.

AUTHOR CONTRIBUTIONS

Conceptualization, D.U. and A.M.; formal analysis, E.P., A.D., J.R., F.R., F.B., L.P., and H.B.; investigation, E.P., A.D., J.R., and A.M.; resources, A.H.; funding, D.U., A.M., J.R., O.K., and L.P.; supervision, D.U., A.M., O.K., and L.P.; writing – original draft, D.U.; writing – review & editing, all authors. All authors have read and agreed to the published version of the manuscript.

DECLARATION OF INTERESTS

The authors declare no competing interests related to this study.

SUPPLEMENTAL INFORMATION

Supplemental information can be found online at https://doi.org/10.1016/j.omton.2024.200903.

REFERENCES

1. Siegel, R.L., Miller, K.D., Fuchs, H.E., and Jemal, A. (2022). Cancer statistics, 2022. CA. Cancer J. Clin. *72*, 7–33.

2. Geistlinger, L., Oh, S., Ramos, M., Schiffer, L., LaRue, R.S., Henzler, C.M., Munro, S.A., Daughters, C., Nelson, A.C., Winterhoff, B.J., et al. (2020). Multiomic analysis of subtype evolution and heterogeneity in high-grade serous ovarian carcinoma. Cancer Res. *80*, 4335–4345.
3. Lheureux, S., Gourley, C., Vergote, I., and Oza, A.M. (2019). Epithelial ovarian cancer. Lancet *393*, 1240–1253.
4. Bell, D., Berchuck, A., Birrer, M., Chien, J., Cramer, D.W., Dao, F., Dhir, R., Disaia, P., Gabra, H., Glenn, P., et al. (2011). Integrated genomic analyses of ovarian carcinoma. Nat *474*, 609–615.
5. Vázquez-García, I., Uhlitz, F., Ceglia, N., Lim, J.L.P., Wu, M., Mohibullah, N., Niyazov, J., Ruiz, A.E.B., Boehm, K.M., Bojilova, V., et al. (2022). Ovarian cancer mutational processes drive site-specific immune evasion. Nat *612*, 778–786.
6. Wang, Y.K., Bashashati, A., Anglesio, M.S., Cochrane, D.R., Grewal, D.S., Ha, G., McPherson, A., Horlings, H.M., Senz, J., Prentice, L.M., et al. (2017). Genomic consequences of aberrant DNA repair mechanisms stratify ovarian cancer histotypes. Nat. Genet. *49*, 856–865.
7. Disis, M.L., Taylor, M.H., Kelly, K., Beck, J.T., Gordon, M., Moore, K.M., Patel, M.R., Chaves, J., Park, H., Mita, A.C., et al. (2019). Efficacy and safety of avelumab for patients with recurrent or refractory ovarian cancer: Phase 1b results from the JAVELIN solid tumor trial. JAMA Oncol. *5*, 393–401.
8. Berek, J.S., Kehoe, S.T., Kumar, L., and Friedlander, M. (2018). Cancer of the ovary, fallopian tube, and peritoneum. Int. J. Gynaecol. Obstet. *143*, 59–78.
9. Okamura, H., and Katabuchi, H. (2005). Pathophysiological dynamics of human ovarian surface epithelial cells in epithelial ovarian carcinogenesis. Int. Rev. Cytol. *242*, 1–54.
10. Sasaki, R., Narisawa-Saito, M., Yugawa, T., Fujita, M., Tashiro, H., Katabuchi, H., and Kiyono, T. (2009). Oncogenic transformation of human ovarian surface epithelial cells with defined cellular oncogenes. Carcinogenesis *30*, 423–431.
11. Domcke, S., Sinha, R., Levine, D.A., Sander, C., and Schultz, N. (2013). Evaluating cell lines as tumour models by comparison of genomic profiles. Nat. Commun. *4*, 2126.
12. Jin, H., Zhang, C., Zwahlen, M., von Feilitzen, K., Karlsson, M., Shi, M., Yuan, M., Song, X., Li, X., Yang, H., et al. (2023). Systematic transcriptional analysis of human cell lines for gene expression landscape and tumor representation. Nat. Commun. *14*, 5417.
13. An, Q., Liu, T., Wang, M.Y., Yang, Y.J., Zhang, Z.D., Liu, Z.J., and Yang, B. (2021). KRT7 promotes epithelial-mesenchymal transition in ovarian cancer via the TGF-β/Smad2/3 signaling pathway. Oncol. Rep. *45*, 481–492.
14. Nonaka, D., Chiriboga, L., and Soslow, R.A. (2008). Expression of Pax8 as a useful marker in distinguishing ovarian carcinomas from mammary carcinomas. Am. J. Surg. Pathol. *32*, 1566–1571.
15. Ortega, M.A., Poirion, O., Zhu, X., Huang, S., Wolfgruber, T.K., Sebra, R., and Garmire, L.X. (2017). Using single-cell multiple omics approaches to resolve tumor heterogeneity. Clin. Transl. Med. *6*, 46.
16. Schubert, M., Klinger, B., Klünemann, M., Sieber, A., Uhlitz, F., Sauer, S., Garnett, M.J., Blüthgen, N., and Saez-Rodriguez, J. (2018). Perturbation-response genes reveal signaling footprints in cancer gene expression. Nat. Commun. *9*, 1–11.
17. Smazynski, J., Hamilton, P.T., Thornton, S., Milne, K., Wouters, M.C.A., Webb, J.R., and Nelson, B.H. (2020). The immune suppressive factors CD155 and PD-L1 show contrasting expression patterns and immune correlates in ovarian and other cancers. Gynecol. Oncol. *158*, 167–177.
18. Bray, M.A., Singh, S., Han, H., Davis, C.T., Borgeson, B., Hartland, C., Kost-Alimova, M., Gustafsdottir, S.M., Gibson, C.C., and Carpenter, A.E. (2016). Cell Painting, a high-content image-based assay for morphological profiling using multiplexed fluorescent dyes. Nat. Protoc. *11*, 1757–1774.
19. Collins, C., and Nelson, W.J. (2015). Running with neighbors: Coordinating cell migration and cell-cell adhesion. Curr. Opin. Cell Biol. *36*, 62–70.
20. Givel, A.M., Kieffer, Y., Scholer-Dahirel, A., Sirven, P., Cardon, M., Pelon, F., Magagna, I., Gentric, G., Costa, A., Bonneau, C., et al. (2018). miR200-regulated CXCL12β promotes fibroblast heterogeneity and immunosuppression in ovarian cancers. Nat. Commun. *9*, 1056.
21. Hu, Z., Artibani, M., Alsaadi, A., Wietek, N., Morotti, M., Shi, T., Zhong, Z., Santana Gonzalez, L., El-Sahhar, S., Carrami, E.M., et al. (2020). The repertoire of serous ovarian cancer non-genetic heterogeneity revealed by single-cell sequencing of normal fallopian tube epithelial cells. Cancer Cell *37*, 226–242.e7.
22. Hu, Y., Pan, J., Shah, P., Ao, M., Thomas, S.N., Liu, Y., Chen, L., Schnaubelt, M., Clark, D.J., Rodriguez, H., et al. (2020). Integrated proteomic and glycoproteomic characterization of human high-grade serous ovarian carcinoma. Cell Rep. *33*, 108276.
23. Hornburg, M., Desbois, M., Lu, S., Guan, Y., Lo, A.A., Kaufman, S., Elrod, A., Lotstein, A., DesRochers, T.M., Munoz-Rodriguez, J.L., et al. (2021). Single-cell dissection of cellular components and interactions shaping the tumor immune phenotypes in ovarian cancer. Cancer Cell *39*, 928–944.e6.
24. Hussain, A., Voisin, V., Poon, S., Karamboulas, C., Bui, N.H.B., Meens, J., Dmytryshyn, J., Ho, V.W., Tang, K.H., Paterson, J., et al. (2020). Distinct fibroblast functional states drive clinical outcomes in ovarian cancer and are regulated by TCF21. J. Exp. Med. *217*, e20191094.
25. Aibar, S., González-Blas, C.B., Moerman, T., Huynh-Thu, V.A., Imrichova, H., Hulselmans, G., Rambow, F., Marine, J.C., Geurts, P., Aerts, J., et al. (2017). SCENIC: Single-cell regulatory network inference and clustering. Nat. Methods *14*, 1083–1086.
26. Wagner, M., Yoshihara, M., Douagi, I., Damdimopoulos, A., Panula, S., Petropoulos, S., Lu, H., Pettersson, K., Palm, K., Katayama, S., et al. (2020). Single-cell analysis of human ovarian cortex identifies distinct cell populations but no oogonial stem cells. Nat. Commun. *11*, 1147.
27. Zannas, A.S., Wiechmann, T., Gassen, N.C., and Binder, E.B. (2016). Gene-stress-epigenetic regulation of FKBP5: Clinical and translational implications. Neuropsychopharmacology *41*, 261–274.
28. Massey, W., Osborn, L.J., Banerjee, R., Horak, A., Fung, K.K., Orabi, D., Chan, E.R., Sangwan, N., Wang, Z., and Brown, J.M. (2022). Flavin-containing monooxygenase 3 (FMO3) is critical for dioxin-induced reorganization of the gut microbiome and host insulin sensitivity. Metabolites *12*, 364.
29. Jia, W., Luo, Q., Wu, J., Shi, Y., and Guan, Q. (2023). Neutrophil elastase as a potential biomarker related to the prognosis of gastric cancer and immune cell infiltration in the tumor immune microenvironment. Sci. Rep. *13*, 13447.
30. Sherwin, J.R.A., Sharkey, A.M., Cameo, P., Mavrogianis, P.M., Catalano, R.D., Edassery, S., and Fazleabas, A.T. (2007). Identification of novel genes regulated by chorionic gonadotropin in baboon endometrium during the window of implantation. Endocrinology *148*, 618–626.
31. Melendez-Zajgla, J., Del Pozo, L., Ceballos, G., and Maldonado, V. (2008). Tissue inhibitor of metalloproteinases-4. The road less traveled. Mol. Cancer *7*, 85.
32. Liu, T.M., Lee, E.H., Lim, B., and Shyh-Chang, N. (2016). Concise review: balancing stem cell self-renewal and differentiation with PLZF. Stem Cell. *34*, 277–287.
33. Yadav, B., Pemovska, T., Szwajda, A., Kulesskiy, E., Kontro, M., Karjalainen, R., Majumder, M.M., Malani, D., Murumägi, A., Knowles, J., et al. (2014). Quantitative scoring of differential drug sensitivity for individually optimized anticancer therapies. Sci. Rep. *4*, 5193.
34. Murumägi, A., Ungureanu, D., Khan, S., Arjama, M., Välimäki, K., Ianevski, A., Ianevski, P., Bergström, R., Dini, A., Kanerva, A., et al. (2023). Drug response profiles in patient-derived cancer cells across histological subtypes of ovarian cancer: real-time therapy tailoring for a patient with low-grade serous carcinoma. Br. J. Cancer *128*, 678–690.
35. Karvonen, H., Arjama, M., Kaleva, L., Niininen, W., Barker, H., Koivisto-Korander, R., Tapper, J., Pakarinen, P., Lassus, H., Loukovaara, M., et al. (2020). Glucocorticoids induce differentiation and chemoresistance in ovarian cancer by promoting ROR1-mediated stemness. Cell Death Dis. *11*, 790.
36. Veneziani, A.C., Gonzalez-Ochoa, E., Alqaisi, H., Madariaga, A., Bhat, G., Rouzbahman, M., Sneha, S., and Oza, A.M. (2023). Heterogeneity and treatment landscape of ovarian carcinoma. Nat. Rev. Clin. Oncol. *20*, 820–842.
37. Zhang, S., Dolgalev, I., Zhang, T., Ran, H., Levine, D.A., and Neel, B.G. (2019). Both fallopian tube and ovarian surface epithelium are cells-of-origin for high-grade serous ovarian carcinoma. Nat. Commun. *10*, 5367.
38. Luo, L., Zhang, W., You, S., Cui, X., Tu, H., Yi, Q., Wu, J., and Liu, O. (2024). The role of epithelial cells in fibrosis: Mechanisms and treatment. Pharmacol. Res. *202*, 107144.

39. Li, X., Wang, F., Xu, X., Zhang, J., and Xu, G. (2021). The dual role of STAT1 in ovarian cancer: insight into molecular mechanisms and application potentials. Front. Cell Dev. Biol. *9*, 636595.

40. Jiang, S., Deng, X., Luo, M., Zhou, L., Chai, J., Tian, C., Yan, Y., and Luo, Z. (2023). Pan-cancer analysis identified OAS1 as a potential prognostic biomarker for multiple tumor types. Front. Oncol. *13*, 1207081.

41. Sudo, S., Avsian-Kretchmer, O., Wang, L.S., and Hsueh, A.J.W. (2004). Protein related to DAN and cerberus is a bone morphogenetic protein antagonist that participates in ovarian paracrine regulation. J. Biol. Chem. *279*, 23134–23141.

42. Myers, M., Tripurani, S.K., Middlebrook, B., Economides, A.N., Canalis, E., and Pangas, S.A. (2011). Loss of gremlin delays primordial follicle assembly but does not affect female fertility in mice. Biol. Reprod. *85*, 1175–1182.

43. Qin, N., Tyasi, T.L., Sun, X., Chen, X., Zhu, H., Zhao, J., and Xu, R. (2020). Determination of the roles of GREM1 gene in granulosa cell proliferation and steroidogenesis of hen ovarian prehierarchical follicles. Theriogenology *151*, 28–40.

44. Kulus, M., Sujka-Kordowska, P., Konwerska, A., Celichowski, P., Kranc, W., Kulus, J., Piotrowska-Kempisty, H., Antosik, P., Bukowska, D., Iżycki, D., et al. (2019). New molecular markers involved in regulation of ovarian granulosa cell morphogenesis, development and differentiation during short-term primary in vitro culture—transcriptomic and histochemical study based on ovaries and individual separated follicles. Int. J. Mol. Sci. *20*, 3966.

45. Wu, X., Zhao, J., Ruan, Y., Sun, L., Xu, C., and Jiang, H. (2018). Sialyltransferase ST3GAL1 promotes cell migration, invasion, and TGF-β1-induced EMT and confers paclitaxel resistance in ovarian cancer. Cell Death Dis. *9*, 1102.

46. Zhang, M., Zhang, Y.Y., Chen, Y., Wang, J., Wang, Q., and Lu, H. (2021). TGF-β signaling and resistance to cancer therapy. Front. Cell Dev. Biol. *9*, 786728.

47. Chen, Z., Han, F., Du, Y., Shi, H., and Zhou, W. (2023). Hypoxic microenvironment in cancer: molecular mechanisms and therapeutic interventions. Signal Transduct. Target. Ther. *8*, 70.

48. Lahtinen, A., Lavikka, K., Virtanen, A., Li, Y., Jamalzadeh, S., Skorda, A., Lauridsen, A.R., Zhang, K., Marchi, G., Isoviita, V.M., et al. (2023). Evolutionary states and trajectories characterized by distinct pathways stratify patients with ovarian high grade serous carcinoma. Cancer Cell *41*, 1103–1117.e12.

49. Hew, K.E., Miller, P.C., El-Ashry, D., Sun, J., Besser, A.H., Ince, T.A., Gu, M., Wei, Z., Zhang, G., Brafford, P., et al. (2016). MAPK activation predicts poor outcome and the MEK inhibitor, selumetinib, reverses antiestrogen resistance in ER-positive high-grade serous ovarian cancer. Clin. Cancer Res. *22*, 935–947.

50. Simpkins, F., Jang, K., Yoon, H., Hew, K.E., Kim, M., Azzam, D.J., Sun, J., Zhao, D., Ince, T.A., Liu, W., et al. (2018). Dual Src and MEK inhibition decreases ovarian cancer growth and targets tumor initiating stem-like cells. Clin. Cancer Res. *24*, 4874–4886.

51. Lee, S., Yoon, S., and Kim, D.H. (2007). A high nuclear basal level of ERK2 phosphorylation contributes to the resistance of cisplatin-resistant human ovarian cancer cells. Gynecol. Oncol. *104*, 338–344.

52. Chesnokov, M.S., Khan, I., Park, Y., Ezell, J., Mehta, G., Yousif, A., Hong, L.J., Buckanovich, R.J., Takahashi, A., and Chefetz, I. (2021). The MEK1/2 pathway as a therapeutic target in high-grade serous ovarian carcinoma. Cancers *13*, 1369.

53. Maeda, T., Tashiro, H., Katabuchi, H., Begum, M., Ohtake, H., Kiyono, T., and Okamura, H. (2005). Establishment of an immortalised human ovarian surface epithelial cell line without chromosomal instability. Br. J. Cancer *93*, 116–123.

54. Bankhead, P., Loughrey, M.B., Fernández, J.A., Dombrowski, Y., McArt, D.G., Dunne, P.D., McQuaid, S., Gray, R.T., Murray, L.J., Coleman, H.G., et al. (2017). QuPath: Open source software for digital pathology image analysis. Sci. Rep. *7*, 1–7.

55. Potdar, S., Ianevski, A., Mpindi, J.P., Bychkov, D., Fiere, C., Ianevski, P., Yadav, B., Wennerberg, K., Aittokallio, T., Kallioniemi, O., et al. (2020). Breeze: An integrated quality control and data analysis application for high-throughput drug screening. Bioinformatics *36*, 3602–3604.

56. Potdar, S., Ianevski, F., Ianevski, A., Tanoli, Z., Wennerberg, K., Seashore-Ludlow, B., Kallioniemi, O., Östling, P., Aittokallio, T., and Saarela, J. (2023). Breeze 2.0: An interactive web-tool for visual analysis and comparison of drug response data. Nucleic Acids Res. *51*, W57–W61.

57. Gu, Z. (2022). Complex heatmap visualization. iMeta *1*, e43.

58. Smith, K., Li, Y., Piccinini, F., Csucs, G., Balazs, C., Bevilacqua, A., and Horvath, P. (2015). CIDRE: An illumination-correction method for optical microscopy. Nat. Methods *12*, 404–406.

59. Hollandi, R., Szkalisity, A., Toth, T., Tasnadi, E., Molnar, C., Mathe, B., Grexa, I., Molnar, J., Balind, A., Gorbe, M., et al. (2020). nucleAIzer: A parameter-free deep learning framework for nucleus segmentation using image style transfer. Cell Syst. *10*, 453–458.e6.

60. Stirling, D.R., Swain-Bowden, M.J., Lucas, A.M., Carpenter, A.E., Cimini, B.A., and Goodman, A. (2021). CellProfiler 4: improvements in speed, utility and usability. BMC Bioinf. *22*, 433.

61. Virtanen, P., Gommers, R., Oliphant, T.E., Haberland, M., Reddy, T., Cournapeau, D., Burovski, E., Peterson, P., Weckesser, W., Bright, J., et al. (2020). SciPy 1.0: fundamental algorithms for scientific computing in Python. Nat. Methods *17*, 261–272.

62. Pedregosa, F., Varoquaux, G., Gramfort, A., Vincent, M., Bertrand, T., Grisel, O., Blondel, M., Prettenhofer, P., Weiss, R., Vincent, D., et al. (2011). Scikit-learn: machine learning in Python. J. Mach. Learn. Res. *12*, 2825–2830.

63. Stoeckius, M., Zheng, S., Houck-Loomis, B., Hao, S., Yeung, B.Z., Mauck, W.M., Smibert, P., and Satija, R. (2018). Cell Hashing with barcoded antibodies enables multiplexing and doublet detection for single cell genomics. Genome Biol. *19*, 224.

64. Hao, Y., Stuart, T., Kowalski, M.H., Choudhary, S., Hoffman, P., Hartman, A., Srivastava, A., Molla, G., Madad, S., Fernandez-Granda, C., and Satija, R. (2024). Dictionary learning for integrative, multimodal and scalable single-cell analysis. Nat. Biotechnol. *42*, 293–304.

65. Tirosh, I., Izar, B., Prakadan, S.M., Wadsworth, M.H., Treacy, D., Trombetta, J.J., Rotem, A., Rodman, C., Lian, C., Murphy, G., et al. (2016). Dissecting the multicellular ecosystem of metastatic melanoma by single-cell RNA-seq. Science *352*, 189–196.

66. Tickle, T., Tirosh, I., Georgescu, C., Brown, M., and Haas, B. (2019). inferCNV of the Trinity CTAT Project (Klarman Cell Observatory, Broad Institute of MIT and Harvard, Cambridge, MA, USA). https://github.com/broadinstitute/inferCNV.

67. Alquicira-Hernandez, J., and Powell, J.E. (2021). Nebulosa recovers single-cell gene expression signals by kernel density estimation. Bioinformatics *37*, 2485–2487.

68. Blighe, K., Rana, S., and Lewis, M. (2024). EnhancedVolcano: Publication-ready volcano plots with enhanced colouring and labeling. R package version 1.24.0. https://github.com/kevinblighe/EnhancedVolcano.

69. Kolberg, L., Raudvere, U., Kuzmin, I., Vilo, J., and Peterson, H. (2020). gprofiler2 – an R package for gene list functional enrichment analysis and namespace conversion toolset g: Profiler. F1000Res. *9*, 709.

70. Wickham, H. (2016). ggplot2: Elegant Graphics for Data Analysis (Springer-Verlag New York), ISBN 978-3-319-24277-4. https://ggplot2.tidyverse.org.

71. Badia-I-Mompel, P., Vélez Santiago, J., Braunger, J., Geiss, C., Dimitrov, D., Müller-Dott, S., Taus, P., Dugourd, A., Holland, C.H., Ramirez Flores, R.O., et al. (2022). decoupleR: ensemble of computational methods to infer biological activities from omics data. Bioinforma. Adv *2*, 1–3.

72. Abdulla, S., Aevermann, B., Assis, P., Badajoz, S., Bell, S.M., Bezzi, E., Cakir, B., Chaffer, J., Chambers, S., Michael Cherry, J., et al. (2023). CZ CELL×GENE Discover: A single-cell data platform for scalable exploration, analysis and modeling of aggregated data. Preprint at bioRxiv. https://doi.org/10.1101/2023.10.30.563174.

73. Lemon, A.J., Bolker, B., Oom, S., Klein, E., Rowlingson, B., Wickham, H., Tyagi, A., Eterradossi, O., Grothendieck, G., Toews, M., et al. (2016). Package 'plotrix'. https://github.com/plotrix/plotrix.

OMTON, Volume *32*

Supplemental information

Molecular and functional profiling of primary normal ovarian cells defines insights into cancer development and drug responses

Emilia Piki, Alice Dini, Frida Rantanen, Franziska Bentz, Lassi Paavolainen, Harlan Barker, Juuli Raivola, Akira Hirasawa, Olli Kallioniemi, Astrid Murumägi, and Daniela Ungureanu

A

Cell line	Source	Histological subtype	Status	Sample type	Key oncogenic aberrations	HR-status (*BRCA1*/*BRCA2*)
HOSE1C	Tashiro Lab (1,2)	Epithelial/mesenchymal (normal tissue)	Non-malignant	Ovarian surface epithelium	CDK4, CyclinD1 overexpression, hTERT	
HOSE2C	Tashiro Lab (1,2)	Epithelial/mesenchymal (normal tissue)	Non-malignant	Ovarian surface epithelium	CDK4, CyclinD1 overexpression, hTERT	
JHOS2	RIKEN	High-grade serous ovarian cancer	Primary	Tissue, ovary	TP53 p.S261_splice, BRCA1 N/A, NF1 p.K798R	*BRCA1* mutation
Kuramochi	JCRB Cell Bank	High-grade serous ovarian cancer	Metastasis	Ascites	TP53 p.D281Y, MYC Amp, KRAS Amp	*BRCA2* mutation
Ovsaho	JCRB Cell Bank	High-grade serous ovarian cancer	Metastasis	Tissue, abdomen	TP53 p.R342X	*BRCA2* homozygous deletion
COV318	ECACC	High-grade serous ovarian cancer	Metastasis	Ascites	TP53 p.I195F	
COV362	ECACC	High-grade serous ovarian cancer	Metastasis	Pleural effusion	TP53 p.Y220C, BRCA1 p.P871fs	*BRCA1* mutation
OV90	ATCC	High-grade serous ovarian cancer	Metastasis	Ascites	TP53 p.S215R, BRAF p.N486-P4990del	
CAOV3	NCI-DTP	High-grade serous ovarian cancer	Primary	Tissue, ovary	TP53 p.Q136*	
OVCAR4	NCI-DTP	High-grade serous ovarian cancer	Metastasis	Ascites	TP53 p.L130V	
OVCAR5	NCI-DTP	High-grade serous ovarian cancer	Metastasis	Ascites	TP53 wt, KRAS p.G12V, CDKN2A homozygous loss	
OVCAR8	NCI-DTP	High-grade serous ovarian cancer	Primary	Tissue, ovary	TP53 p.Y126_K132del, ERBB2 p.G776V	

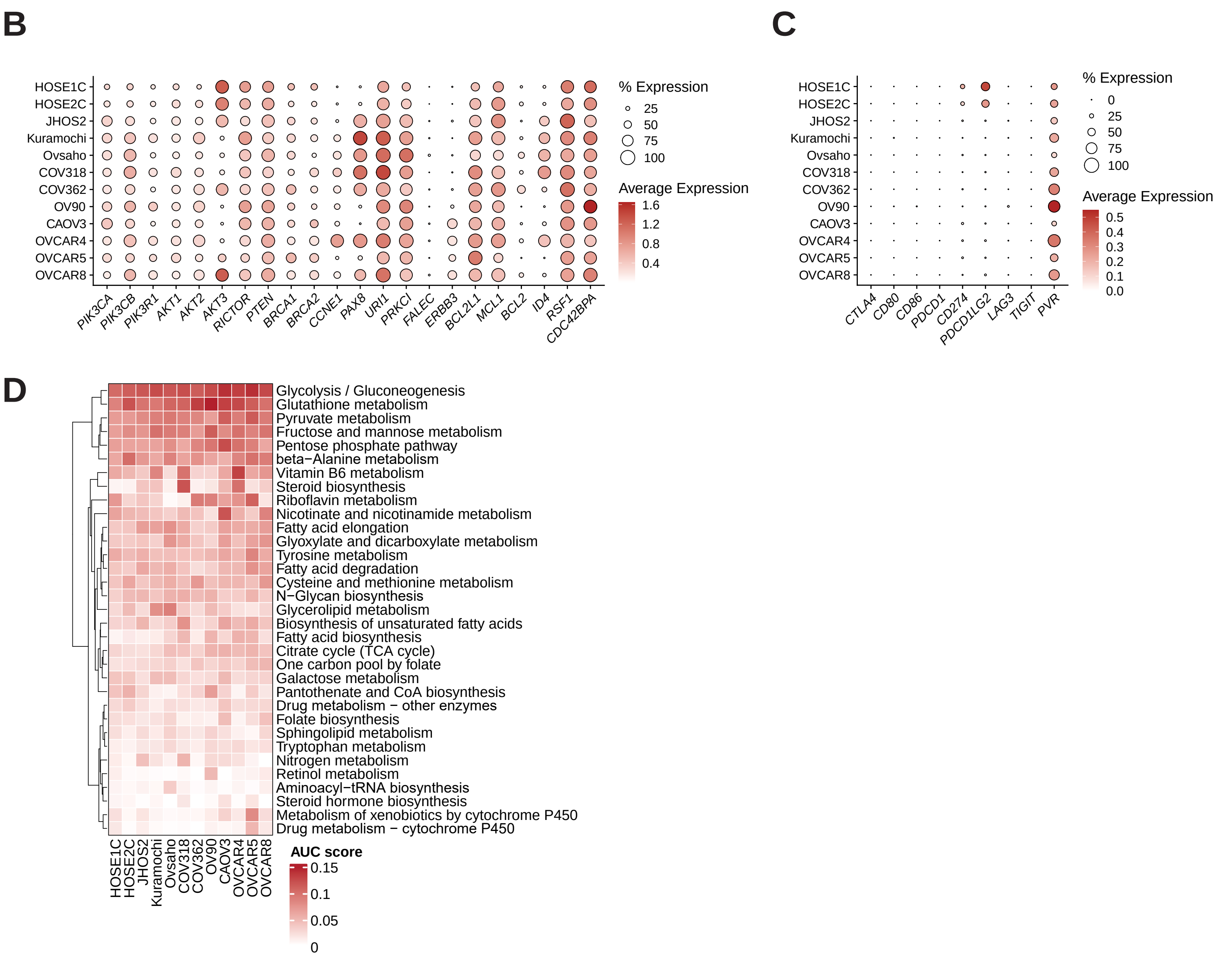

Figure S1

(A) A table presenting the sources of cell lines, their histological subtypes, (disease) status, tissue origin and the key oncogenic aberrations (1 PMID: 15956975, 2 PMID: 19126650).
(B) Expression levels of the relevant targetable genes for two HOSE and ten HGSOC cell lines (PMID: 35582310, PMID: 36804485).
(C) Expression levels of positive immune checkpoints and their ligands for two HOSE and ten HGSOC cell lines (PMID: 22437870, PMID: 23868869).
(D) A heatmap displaying the activity of key metabolic pathways for two HOSE and ten HGSOC cell lines. Pathway activity was quantified using AUC scores.

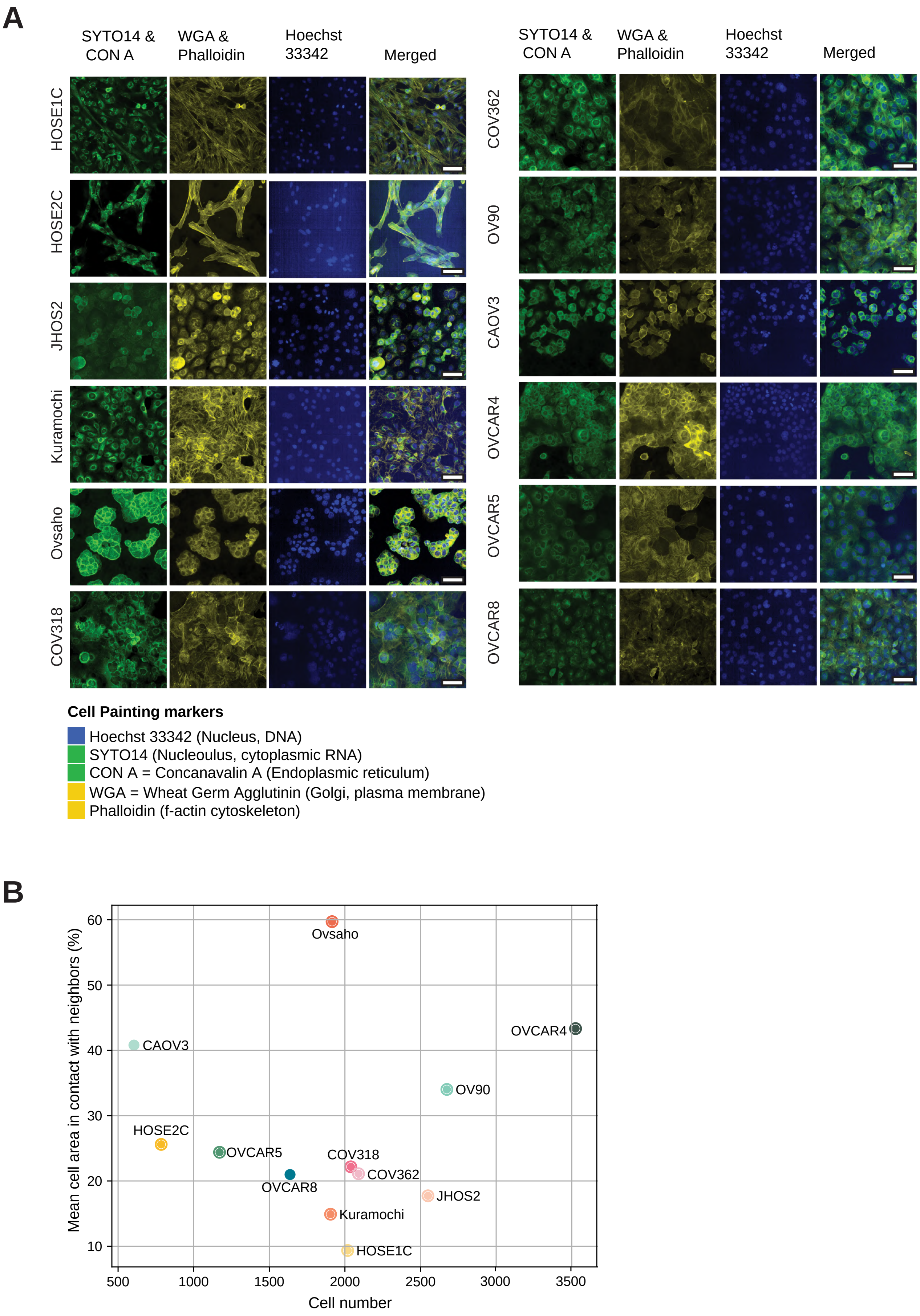

Figure S2

(A) Representative confocal images of Cell Painting of ten HGOC and two HOSE cell lines. The Cell Painting markers used in the assay and the cellular compartments they visualize indicated on the bottom. Scale bar 100 µm.
(B) Relative (%) average mean cell area in contact with neighbor cells based on Cell Painting analysis. X-axis reports average cell number in images.

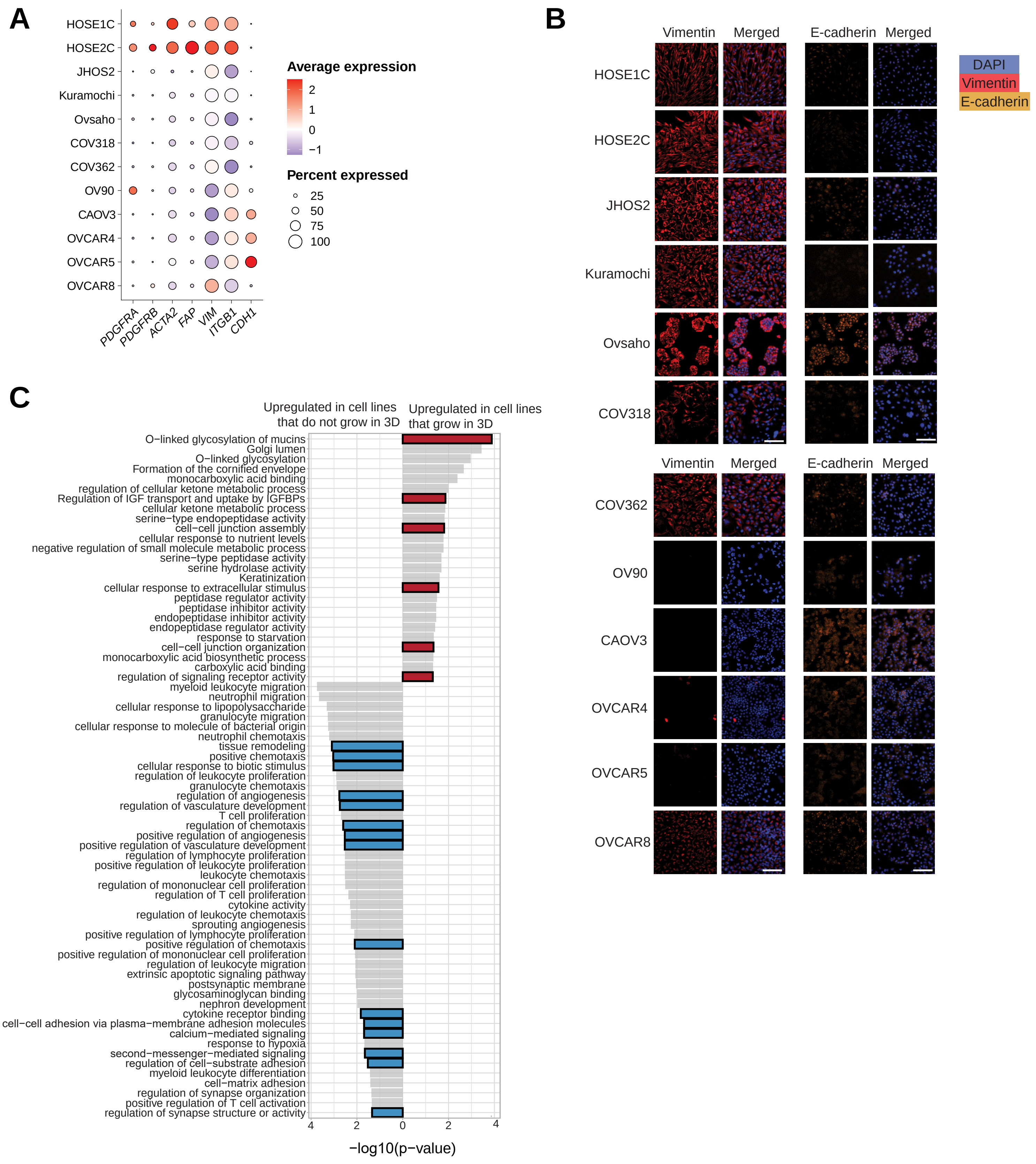

Figure S3

(A) Dot plot illustrating the fraction of cell lines expressing the selected genes (size of dot) and the intensity of expression (color shading). Average expression is scaled.
(B) Immunofluorescence staining of vimentin (in red) and E-cadherin (in orange) of ten HGSOC and two HOSE cell lines. DAPI (in blue) was used as nuclear staining. Scale bar: 100 µm.
(C) Waterfall plot depicting the most upregulated pathways on transcriptomic level in HGSOC cell lines grouped based on their abliity to grow in 3D. Highlighted in colors are pathways selected based on their relevance to OC (related to Figure 3D).

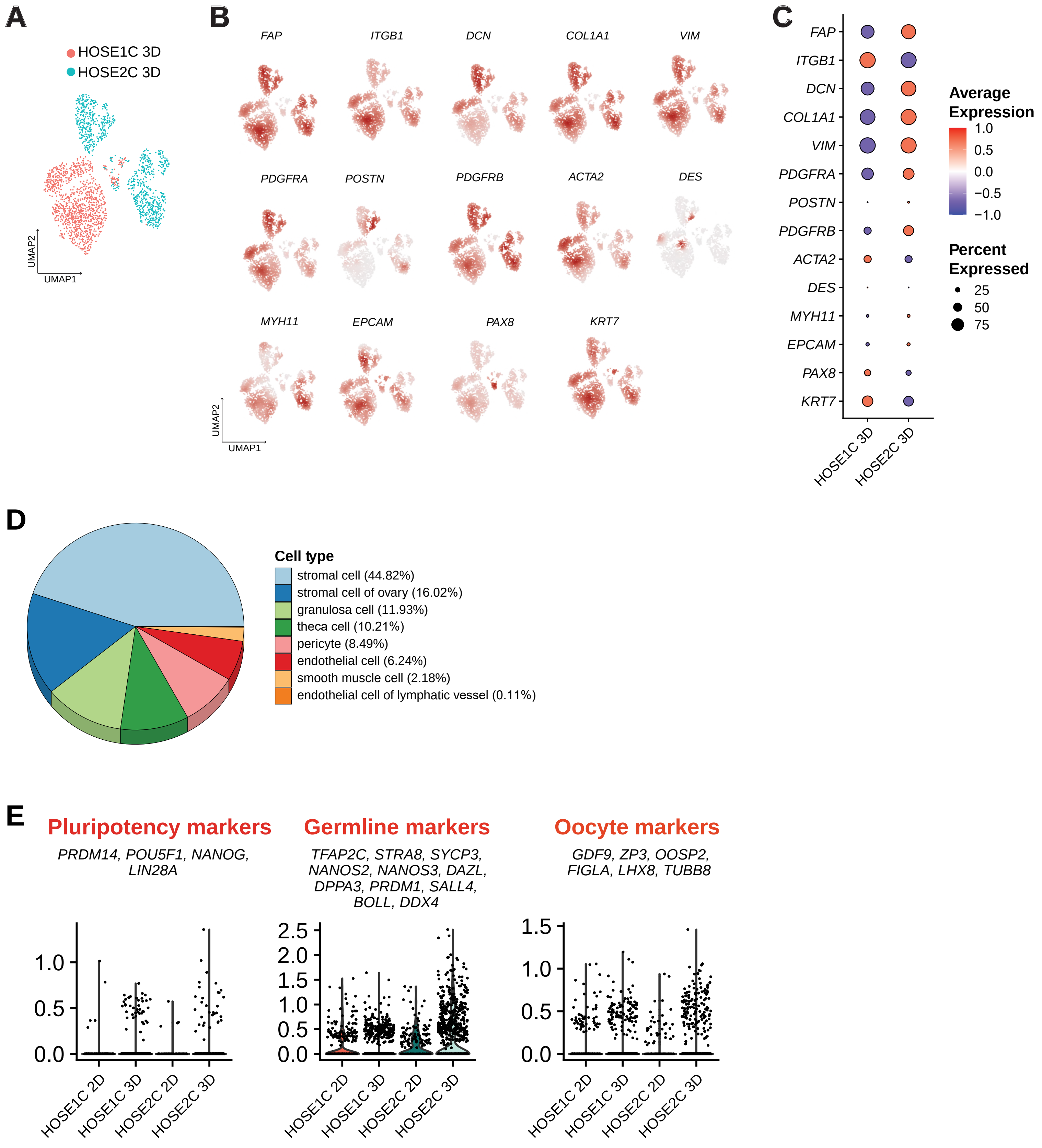

Figure S4

(A) UMAP plot of HOSE1C and HOSE2C cells grown 3D.
(B) UMAP showing the mRNA expression of selected genes in HOSE1C and HOSE2C grown in 3D.
(C) Dot plot illustrating the fraction of cell lines expressing the selected genes (size of dot) and the intensity of expression (color shading).
(D) Pie chart representing the fractions of different ovarian cell subtypes in the Figure 3G, comprising of 39.954 single cells of ovary from CellXGene dataset.
(E) The expression of selected pluripotency, germline and oocyte marker genes in HOSE1C and HOSE2C grown in 2D and 3D.

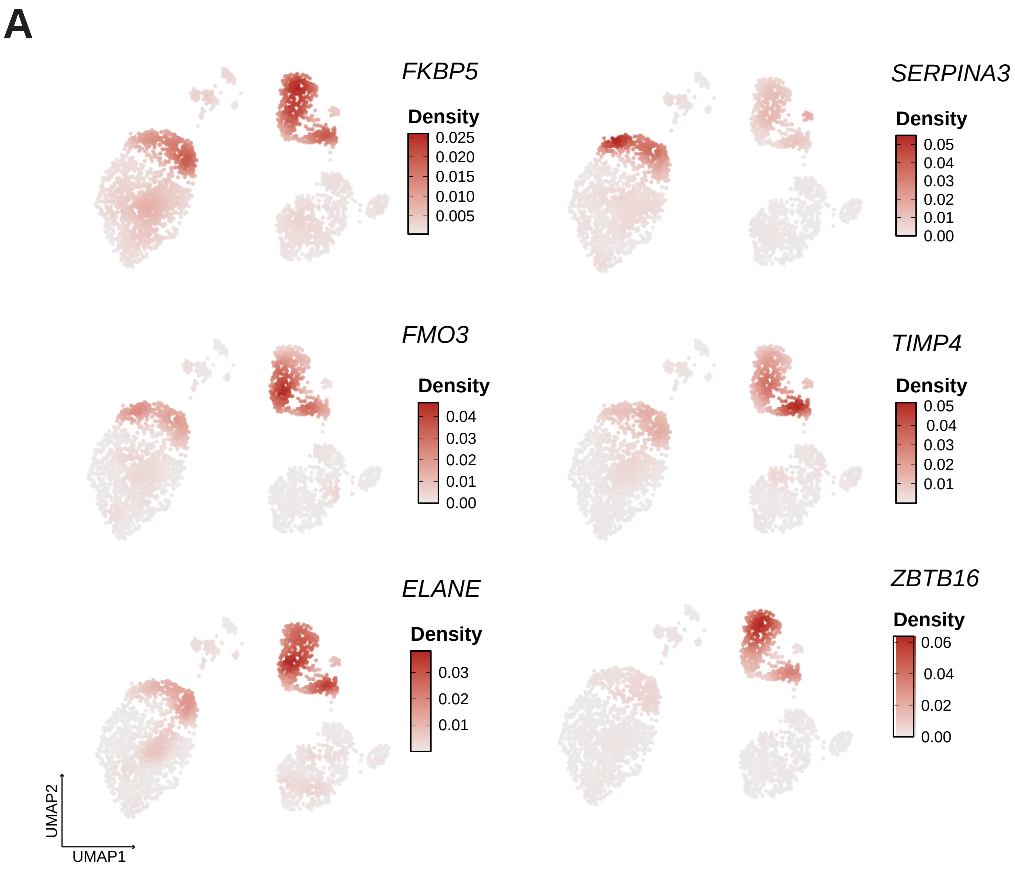

Figure S5

(A) UMAPs showing the mRNA expression of selected genes in untreated and cancer cell- derived condition media (CM) treated HOSE cells in 3D setting. Clustering is presented in Figure 5A.

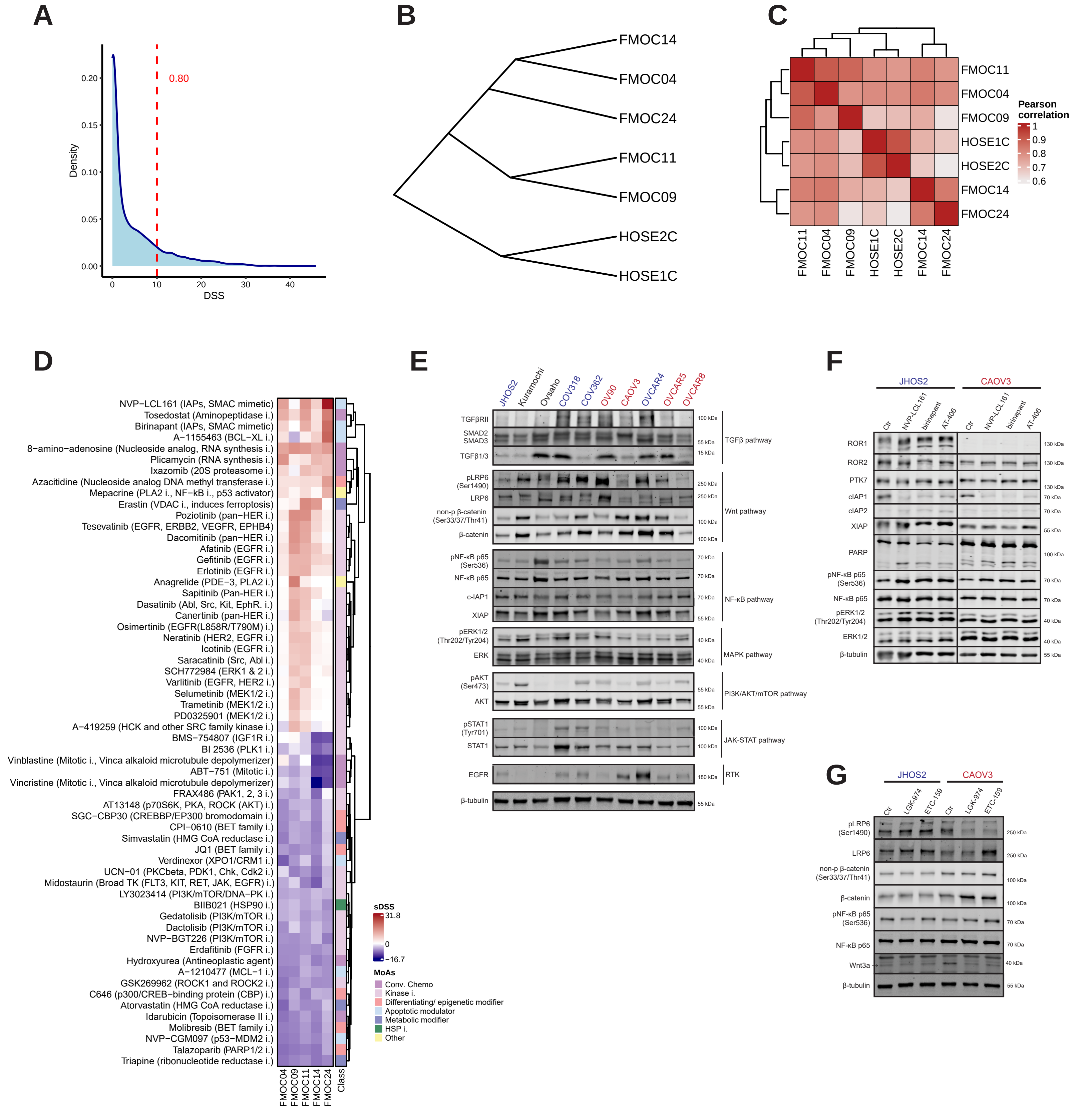

Figure S6

(A) Overall distribution of DSS values across ten OC and two HOSE cell lines. A DSS ≥ 10 (corresponding to the 80th percentile) was chosen as the sensitivity threshold.
(B) Cladogram showing the complete linkage hierarchical clustering results of the five HGSOC PDCs from Murumägi et al., (PMID:36476658) and two HOSEs based on their sensitivity to the 503 drugs included in the DSRT screen.
(C) Pearson correlation among the PDCs and the HOSE cell lines based on their sensitivity to the 503 drugs.
(D) Heatmap showcasing the sDSS for individual drugs. The sDSS was derived by subtracting the average DSS of HOSE1C and HOSE2C from the respective DSSs of PDCs. A subset of the 60 most extreme drugs (top and bottom 30 from the clustered heatmap in Table S1) has been highlighted.
(E) Immunoblot analysis showing the expression of selected markers of TGFβ, Wnt, NF-κB, MAPK, PI3K/AKT/mTOR and JAK-STAT pathways in cell lines lysates.
(F) Immunoblot analysis of JHOS2 and CAOV3 cells treated with SMAC mimetics (NVP-LC161, birinapant and AT-406).
(G) Immunoblot analysis of JHOS2 and CAOV3 cells treated with Porcupine inhibitors (LGK-974 and ETC-159).

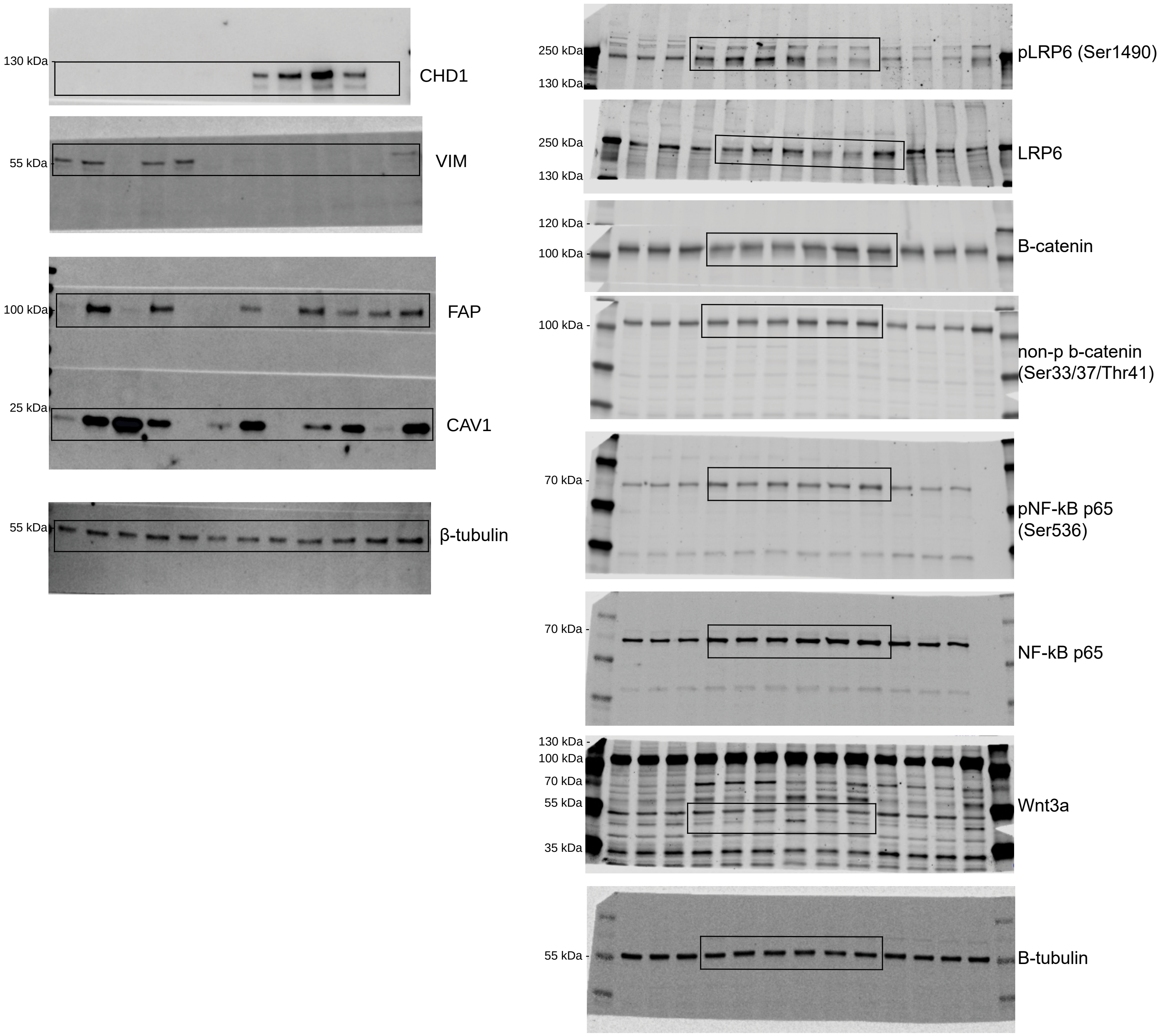

Figure S7

Uncropped images of the immuno blots presented in Figure 3B (left) and in Supplementary Figure 6G (right).

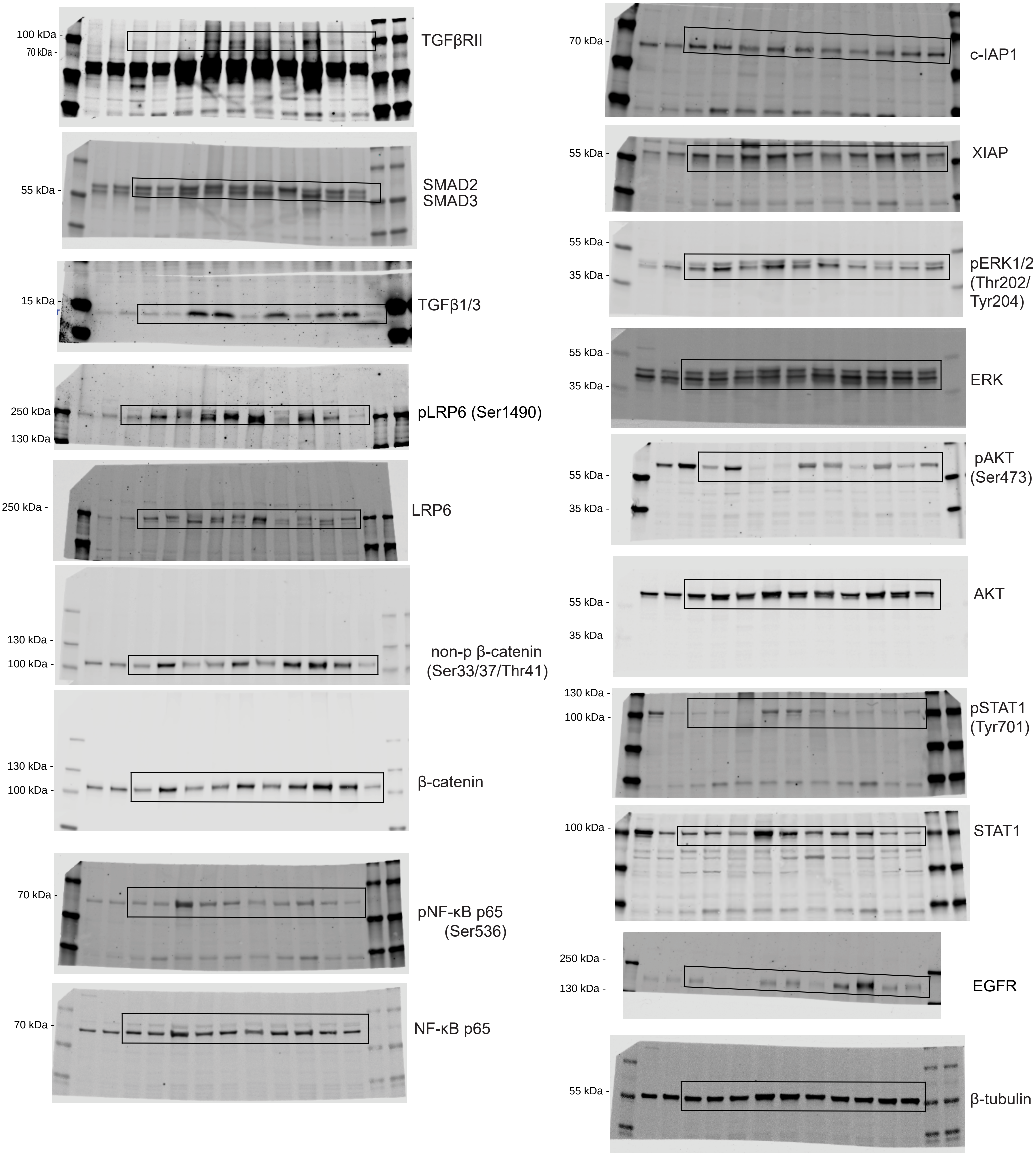

Figure S7

Uncropped images of the immuno blots presented in Supplementary Figure 6E.

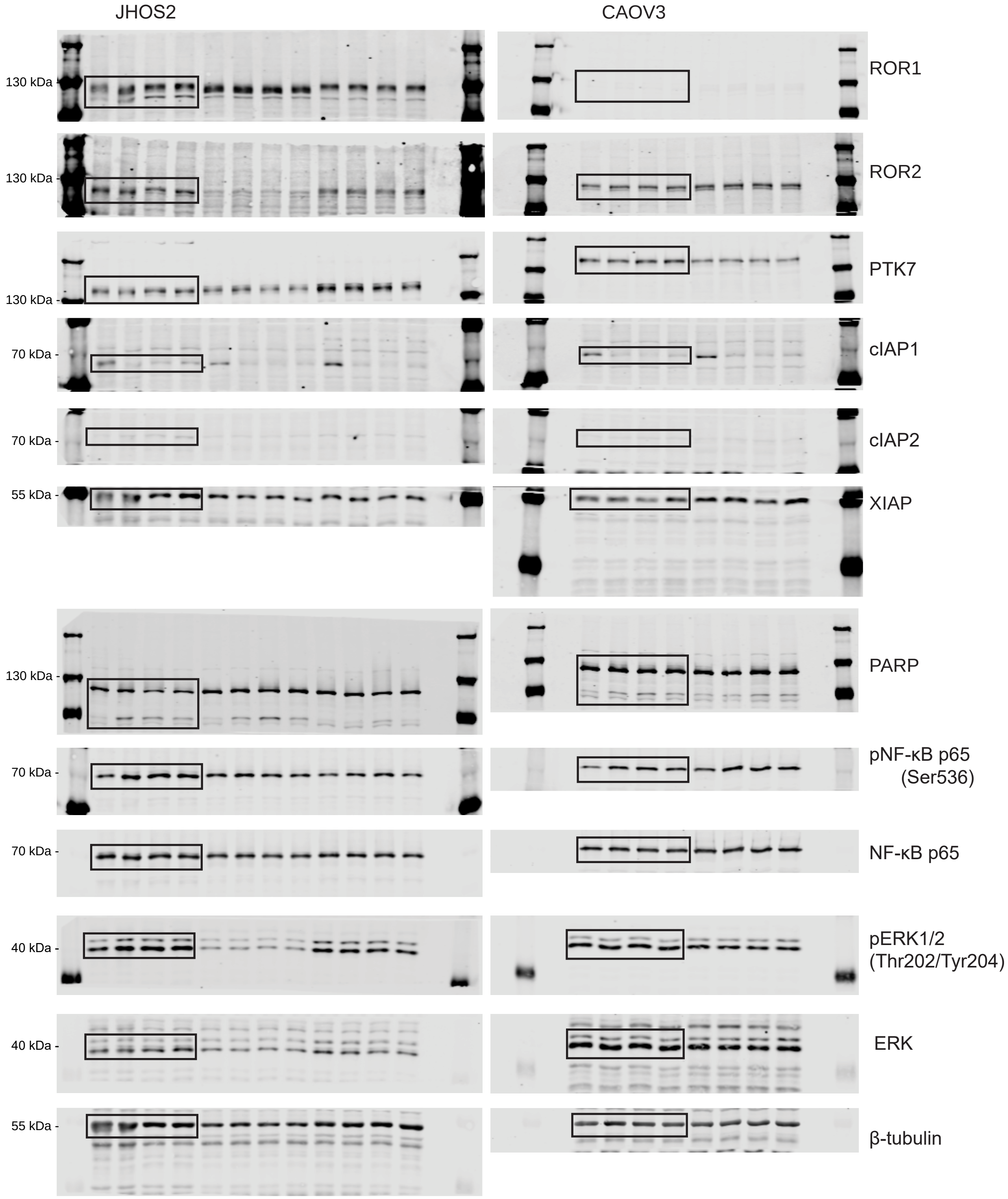

Figure S7

Uncropped images of the immuno blots presented in Supplementary Figure 6F.

Supplemental Tables

Table S1. Drug sensitivity score (DSS) data for all 12 cell lines and 5 PDCs. Data is shown for 10 HGSOC cell lines, 2 HOSE cell lines and 5 patient-derived cancer cells screened with the panel of 503 drugs.

Table S2. Key resources table. For the staining and immunoblotting, the information of the dye and antibody dilutions used in immunofluorescence (IF), immunoblotting (WB) and Cell Painting are indicated in the notes.

REAGENT or RESOURCE	SOURCE	IDENTIFIER	NOTES
Antibodies			
AKT	Cell Signaling Technology (CST)	Cat# 2920; RRID:AB_1147620	WB, 1:1000.
$\beta_2$microglobulin	BioLegend	Cat# 316302; RRID:AB_492835	
β-catenin	Cell Signaling Technology (CST)	Cat# 8480; RRID:AB_11127855	WB, 1:1000.
β-tubulin	Cell Signaling Technology (CST)	Cat# 86298; RRID:AB_2715541	WB, 1:1000.
Caveolin-1 (D46G3)	Cell Signaling Technology (CST)	Cat# 3267; RRID:AB_2275453	WB, 1:1000.
c-IAP1	Cell Signaling Technology (CST)	Cat# 7065; RRID:AB_10890862	WB, 1:1000.
c-IAP2	Cell Signaling Technology (CST)	Cat# 3130; RRID:AB_10693298	WB, 1:1000.
CD298	BioLegend	Cat# 341712; RRID:AB_2876646	
E-Cadherin (24E10)	Cell Signaling Technology (CST)	Cat# 3195; RRID:AB_2893074	IF, 1:100. WB, 1:100.
EGFR	Cell Signaling Technology (CST)	Cat# 4267; RRID:AB_2895042	WB, 1:1000.
ERK	Cell Signaling Technology (CST)	Cat# 4696; RRID:AB_390780	WB, 1:1000.
FAP (EPR20021)	Abcam	Cat# ab207178; RRID:AB_2864720	WB, 1:500.

IRDye® 680RD Donkey anti-Rabbit IgG	LI-COR	Cat# 926-68073; RRID: AB_10954442	
IRDye® 800CW Donkey anti-Mouse IgG	LI-COR	Cat# 926-32212; RRID: AB_621847	
LRP6	Cell Signaling Technology (CST)	Cat# 3395; RRID:AB_1950408	WB, 1:500.
NF-KB p65	Cell Signaling Technology (CST)	Cat# 6956; RRID:AB_10828935	WB, 1:1000.
non-p β-catenin	Cell Signaling Technology (CST)	Cat# 8814; RRID:AB_11127203	WB, 1:1000.
pAKT (Ser473)	Cell Signaling Technology (CST)	Cat# 4060; RRID:AB_2315049	WB, 1:1000.
pERK1/2 (Thr202/Tyr204)	Cell Signaling Technology (CST)	Cat# 4370; RRID:AB_2315112	WB, 1:1000.
PARP	Cell Signaling Technology (CST)	Cat# 9532; RRID:AB_659884	WB, 1:1000.
pLRP6 (Ser1490)	Cell Signaling Technology (CST)	Cat# 2568; RRID:AB_2139327	WB, 1:250.
pNF-KB p65 (Ser536)	Cell Signaling Technology (CST)	Cat# 3033; RRID:AB_331284	WB, 1:1000.
pSTAT1 (Tyr701)	Cell Signaling Technology (CST)	Cat# 88845	WB, 1:1000.
PTK7	Cell Signaling Technology (CST)	Cat# 25618; RRID:AB_2798907	WB, 1:1000.
ROR1	Cell Signaling Technology (CST)	Cat# 16540; RRID:AB_2798764	WB, 1:1000.
ROR2	Cell Signaling Technology (CST)	Cat# 88639; RRID:AB_2800126	WB, 1:1000.
SMAD2/3	Cell Signaling Technology (CST)	Cat# 8685; RRID:AB_10889933	WB, 1:1000.
STAT1	Cell Signaling Technology (CST)	Cat# 65917	WB, 1:1000.
TGFβ1/3	Cell Signaling Technology (CST)	Cat# 3709; RRID:AB_2063357	WB, 1:500.
TGFβRII	Cell Signaling Technology (CST)	Cat# 41896	WB, 1:1000.

Vimentin	Cell Signaling Technology (CST)	Cat# 5741; RRID:AB_10695459	IF, 1:100. WB, 1:1000.
Wnt3a	Cell Signaling Technology (CST)	Cat# 2721; RRID:AB_2215411	WB, 1:500.
XIAP	Cell Signaling Technology (CST)	Cat# 2045; RRID:AB_2214866	WB, 1:1000.
Chemicals, peptides, and recombinant proteins			
Blocker™ BSA	Thermo Fisher Scientific	Cat# 37525	
Concanavalin A	Thermo Fisher Scientific	Cat# C11252	Cell Painting, 1:50, incubation 20min at RT, excitation 488 nm.
DAPI	Sigma-Aldrich	Cat# D9542	
Gibco™ DMEM	Thermo Fisher Scientific	Cat# 41965039	
Gibco™ DMEM/F-12, no glutamine	Thermo Fisher Scientific	Cat# 21331020	
Gibco™ fetal bovine serum	Thermo Fisher Scientific	Cat# 11573397	
Gibco™ HPLM media	Thermo Fisher Scientific	Cat# A4899101	
Gibco™ MEM Non-Essential Amino Acids Solution	Thermo Fisher Scientific	Cat# 11140050	
Gibco™ RPMI 1640	Thermo Fisher Scientific	Cat# 31870025	
Gibco™ TrypLE Express Enzyme	Thermo Fisher Scientific	Cat# 12605010	
Hoechst 33342	Thermo Fisher Scientific	Cat# 62249	Cell Painting, 1:5000, incubation 20min at RT, excitation 405 nm.
L-Glutamine	Sigma-Aldrich	Cat# G7513	
Phalloidin 568	Thermo Fisher Scientific	A12380	Cell Painting, 1:50, incubation 20min at RT, excitation 561 nm.
Primocin®	Invivogen	Cat# ant-pm-2	
Phosphatase Inhibitor Cocktail	Bimake.com	B15001	
SYTO14	Thermo Fisher Scientific	Cat# S7576	Cell Painting, 1:500, incubation 20min at RT, excitation 488 nm.
Triton™ X-100	Sigma-Aldrich	Cat# X100-100ML	

VitroGel ORGANOID-4	TheWell Bioscience	Cat# VHM04-4	
Wheat Germ Agglutinin	Thermo Fisher Scientific	Cat# W32464	Cell Painting, 1:500, incubation 20min at RT, excitation 561nm.
2x Laemmli Sample Buffer	Bio-RAD	Cat# 1610737	
4–20% Mini-PROTEAN® TGX Stain-Free™ Protein Gels	Bio-RAD	Cat# 4568096	
Critical commercial assays			
Chromium Next GEM Single Cell 3' Gene Expression version 3.1 Dual Index chemistry with Feature Barcoding	10x Genomics, FIMM Single-Cell Analytics unit	Cat# CG000317	
CellTiter-Glo® 2.0 Assay	Promega	Cat# G9242	
Deposited data			
DSRT data	This paper		
Experimental models: Cell lines			
COV318	European Collection of Authenticated Cell Cultures (ECACC)	RRID:CVCL_2419	
COV362	European Collection of Authenticated Cell Cultures (ECACC)	RRID:CVCL_2420	
HOSE1C	Tashiro Lab	N/A	
HOSE2C	Tashiro Lab	N/A	
JHOS2	Riken BioResource Research Center (BRC)	RRID:CVCL_4647	
Kuramochi	Japanese Collection of Research Bioresources (JCRB)	RRID:CVCL_1345	
CAOV3	National Cancer Institute - Developmental Therapeutics Program (NCI-DTP)	RRID:CVCL_0201	
OVCAR4	National Cancer Institute - Developmental Therapeutics Program (NCI-DTP)	RRID:CVCL_1627	

OVCAR5	National Cancer Institute - Developmental Therapeutics Program (NCI-DTP)	RRID:CVCL_1628	
OVCAR8	National Cancer Institute - Developmental Therapeutics Program (NCI-DTP)	RRID:CVCL_1629	
Ovsaho	Japanese Collection of Research Bioresources (JCRB)	RRID:CVCL_3114	
OV90	American Type Culture Collection (ATCC)	RRID:CVCL_3768	
Software and algorithms			
QuPath (0.4.3)	Bankhead et al	RRID:SCR_018257	
Image Studio™ Lite (5.2)	LI-COR	RRID:SCR_013715	
ZEN Digital Imaging for Light Microscopy	Carl Zeiss AG	RRID:SCR_013672	
R	R Core Team	RRID:SCR_001905	
CIDRE	Smith et al	N/A	
nucleAIzer	Hollandi et al	N/A	
Cell Profiler (4.2.5)	Stirling et al	RRID:SCR_007358	
Cell Ranger (7.1.0)	10x Genomics	RRID:SCR_017344	
Seurat (5.0.1)	Hao and Hao et al	RRID:SCR_016341	
InferCNV package (1.18.1)	Tickle et al	RRID: SCR_021140	
gprofiler2 package (0.2.2)	Kolberg et al	RRID:SCR_018190	
decoupleR (2.8.0)	Badia-i-Mompel et al	N/A	
PROGENy	Schubert et al	N/A	
pySCENIC (0.12.1)	Aibar et al	RRID: SCR_017247	
BREEZE	Potdar et al	N/A	
GraphPad Prism (9.3.1)	GraphPad Prism Software, LLC	RRID: SCR_002798	
Harmony (0.1.1)	Korsunsky et al	RRID:SCR_022206	
Leiden algorithm	Traag et al	N/A	
